# Polymorphonuclear Leukocytes or Hydrogen Peroxide Enhance Biofilm Development of Mucoid *Pseudomonas aeruginosa*

**DOI:** 10.1155/2018/8151362

**Published:** 2018-07-04

**Authors:** Qi Tan, Qing Ai, Qi Xu, Fang Li, Jialin Yu

**Affiliations:** ^1^Department of Neonatology, Children's Hospital of Chongqing Medical University, Chongqing, China; ^2^Chongqing Key Laboratory of Pediatrics, Chongqing, China; ^3^Ministry of Education Key Laboratory of Child Development and Disorders, Chongqing, China; ^4^China International Science and Technology Cooperation Base of Child Development and Critical Disorders, Chongqing, China; ^5^Department of Pediatric, The Affiliated Hospital of Shenzhen University, Shenzhen, Guangdong, China

## Abstract

*Pseudomonas aeruginosa* is an opportunistic pathogenic bacterium involved in many human infections, including pneumonia, diabetic foot ulcers, and ventilator-associated pneumonia. *P. aeruginosa* cells usually undergo mucoid conversion during chronic lung infection in patients with cystic fibrosis (CF) and resist destruction by polymorphonuclear leukocytes (PMNs), which release free oxygen radicals (ROS), such as H_2_O_2_. PMNs are the main leucocytes in the CF sputum of patients who are infected with *P. aeruginosa*, which usually forms biofilms. Here, we report that PMNs or H_2_O_2_ can promote biofilm formation by mucoid *P. aeruginosa* FRD1 with the use of the hanging-peg method. The mucoid strain infecting CF patients overproduces alginate. In this study, PMNs and H_2_O_2_ promoted alginate production, and biofilms treated with PMNs or H_2_O_2_ exhibited higher expression of alginate genes. Additionally, PMNs increased the activity of GDP-mannose dehydrogenase, which is the key enzyme in alginate biosynthesis. Our results demonstrate that PMNs or H_2_O_2_ can enhance mucoid *P. aeruginosa* biofilms.

## 1. Introduction

*Pseudomonas aeruginosa*, an opportunistic pathogen, usually invades patients who are immunocompromised or immunodeficient. Persistent infection by *P. aeruginosa* was identified to be the main cause of morbidity and mortality in patients with cystic fibrosis (CF) [1, 2]. Following persistent infection, *P. aeruginosa* undergoes significant phenotypic and genetic changes to adapt to airways in chronic CF, including mucoid conversion, and decreases in virulence factor expression and biofilm formation [3, 4]. A biofilm, which is a special arrangement of bacteria, is formed of bacterial cells embedded within an extracellular matrix of polysaccharides produced by the bacteria. Bacteria in biofilms exhibit greater resistance to antibiotics and host defense systems than bacteria growing in planktonic cultures [5, 6].

Polymorphonuclear leukocytes (PMNs) are phagocytic cells that produce a wide range of antimicrobial agents aimed at killing invading bacteria. Chronic *P. aeruginosa* infections have commonly been characterized by the presence of many surrounding PMNs [7]. However, PMNs cannot remove *P. aeruginosa* biofilms and release their dangerous antimicrobial materials into the airway lumen, contributing to tissue damage [8]. The presence of PMNs can upregulate the synthesis of some QS-controlled virulence factors, including rhamnolipids in wild-type *P. aeruginosa* [9], and the inhibition of rhamnolipid synthesis in *P. aeruginosa* by inactivation of the rhamnolipid *rhlA* gene-disabled bacterial protection against PMNs [10]. Some studies have also demonstrated that PMNs could promote biofilm formation by PAO1, allowing it to resist eradication. For example, Walker et al. reported that PMNs could enhance the initial development of biofilms, because polymers composed of actin and DNA bound PAO1, and the number of viable PAO1 cells in the biofilm significantly increased with the presence of PMNs [11]. Mathee et al. reported that reactive oxygen species (ROS) released from activated PMNs could facilitate the generation of mucoid variants during wild-type *P. aeruginosa* infection in the CF airway environment [12]. Thus, mucoid conversion and biofilm formation make *P. aeruginosa* resistant to most PMN antimicrobial effector mechanisms.

Wild-type *P. aeruginosa* has been the main focus of studies during the past decades. However, previous studies reported that approximately 85% of *P. aeruginosa* strains that were isolated from the lungs of CF patients have mucoid colony morphology, and this morphology is more common in the strains isolated from patients in the advanced stages of CF [3, 13]. The typical mucoid phenotype is caused by the overproduction of alginate, and alginate has functions in persistence and immune evasion from PMNs [14]. However, the effects of PMNs on mucoid *P. aeruginosa* biofilms have not been studied. We explored the effects of PMNs or H_2_O_2_ on the biofilm of mucoid *P. aeruginosa* FRD1 and alginate production in vitro. This work may provide a new insight into the mechanism of persistent infection caused by mucoid *P. aeruginosa*.

## 2. Materials and Methods

### 2.1. Bacterial Strain, Media, and Culture Conditions

*P. aeruginosa* FRD1 (CF isolate, mucA, Ohman and Chakrabarty, 1981) was used in this study. Bacteria from frozen stocks were plated on Luria-Bertani (LB) agar (Sigma, St. Louis, MO) and then inoculated into LB liquid medium, which was incubated at 37°C with agitation (200 rpm). FRD1 biofilms were fostered in Jensen's chemically defined medium at 37°C [15].

### 2.2. Isolation of PMNs

PMNs were isolated from human peripheral blood from normal healthy adults who had read and signed donor consent forms. The plasma Percoll method was used for PMN isolation, as described elsewhere [16], and PMNs were resuspended in RPMI 1640 with 10% fetal bovine serum. The obtained cell suspensions contained more than 95% PMNs, and the use of trypan blue (0.4%) showed that their viability was greater than 95%. This study was carried out in accordance with the recommendations of the Medical Ethics Committees of Chongqing Medical University with written informed consent from all subjects. All subjects gave written informed consent in accordance with the Declaration of Helsinki. The protocol was approved by the Medical Ethics Committees of Chongqing Medical University.

### 2.3. Biofilm Assays

FRD1 biofilms were grown by using the previously described hanging-peg method with a small improvement [17]. Briefly, a device containing 96 polystyrene pegs (catalog number 445497; Nunc) was hung in a microtiter plate (catalog number 269787; Nunc). To form biofilms, the pegs were placed in a sterile 96-well plate that had been filled with Jensen's chemically defined medium and bacteria (OD600 = 0.1), and the whole assembly was then incubated at 37°C. The medium was refreshed every day. After initial attachment, then the biofilm forms multicellular structures (“microcolonies”) after the first day of culture, which was early biofilm in this assay, and the maturation of microcolonies goes into thick, three-dimensional structures encased in an exopolymeric matrix after the third day of culture, which was a mature biofilm in this assay [5]. The FRD1 biofilms were stimulated by PMN suspensions (activated with 100 ng/ml phorbol 12-myristate 13-acetate (PMA) and concomitantly during the incubation of PMNs with the biofilm, 2 × 10^5^ cells/peg), H_2_O_2_ (1 mM or 2 mM), and doubly distilled water (without PMA) for 120 min every time, three times per day [12]. In addition, a biofilm of FRD1 was treated with 100 ng/ml PMA or nonactivated PMNs for 120 min every time, three times per day.

PMNs and H_2_O_2_ were removed after the treatment, and the biofilms were moved into fresh medium and thus keep on growing. Because PMNs are continuously recruited to the CF airway [18], the effect of PMNs on biofilms was tested for one or two days. After biofilms were treated with activated PMNs or H_2_O_2_, the peg lids were removed and rinsed three times with sterile phosphate-buffered saline (PBS) and then moved into fresh PBS to incubate in a water bath sonicator (Tomy UD-201, Tokyo, Japan) for 20 min. The concentration of PMNs (2 × 10^6^/ml or 2 × 10^5^/peg) that was used in this analysis was based on bronchoalveolar lavage sampling of the airways of CF patients with persistent *P. aeruginosa* infections; under these conditions, the concentration of PMNs ranged from 10^4^ to 10^6^ per milliliter [19].

To determine the bacterial counts by the plate counting method, we collected cells from parallel pegs after they were treated with activated PMNs or H_2_O_2_ (as described previously) and then plated them on LB agar after they were serially diluted. The bacterial counts were determined after 18 hours of incubation at 37°C.

### 2.4. Adhesion Assays

Considering that alginate interferes with the adhesion of mucoid *P. aeruginosa* [20] and the mucoid *P. aeruginosa* FRD1 in our experiment could not attach to the pegs well in the first several hours (data not shown), we tested the effects of PMNs or H_2_O_2_ on the adhesion of FRD1 during the first day of culture. To enable bacterial adhesion, we fostered FRD1 in Jensen's chemically defined medium using the hanging-peg method described above. After the bacteria were treated with activated PMNs or H_2_O_2_ for 1 day, the pegs were washed three times with sterile PBS. The pegs were then incubated with crystal violet (100 *μ*l, Beyotime, China) for 10 min and washed three times with PBS. Finally, the crystal violet was dissolved with ethanol, and the absorbance at 595 nm was measured using a microplate reader.

### 2.5. Alginate Assays

Biofilms were cultured in the manner described above, with or without activated PMN or H_2_O_2_ treatments. Alginates were collected from cultures grown in 10 ml LB in which the bacteria were from one peg, with rapid aeration at 37°C for 18 h, as described elsewhere [21–24]. The levels of alginate in culture medium were determined as described by Knutson and Jeanes, with some modifications [25]. Briefly, samples (10 ml) of cultures were mixed with 10 ml of saline, and the cells were removed by centrifugation (12,000*g* for 30 min at 4°C). The supernatant was mixed with 2% cetylpyridinium chloride (10 ml), and the alginate precipitate was collected by centrifugation (12,000*g* for 10 min at room temperature). The alginate was purified by dissolving it in NaCl (10 ml, 1 M) and then precipitating with cold (−20°C) isopropanol (10 mL); this purification procedure was repeated once. Finally, the concentration of alginate in solution was determined using the carbazole method [26]. Briefly, alginate solution (30 *μ*l) was first mixed with 1.0 ml of borate-sulfuric acid reagent (10 mM H_3_BO_3_ in concentrated H_2_SO_4_) and 300 *μ*l of carbazole reagent (0.1% in ethanol). Then, the mixture was incubated in a 55°C bath for 30 min, and the absorbance at 530 nm was determined spectrophotometrically. The alginate concentration was calculated by extrapolation from a standard curve that was constructed using a range of alginate concentrations (Sigma).

### 2.6. Real Time PCR (RT-PCR)

The expression of genes related to alginate biosynthesis was investigated using reverse transcriptase-polymerase chain reaction (RT-PCR). Biofilms were cultured in the manner described above, with or without activated PMN or H_2_O_2_ treatments. The peg lids were incubated in a water bath sonicator (Tomy UD-201, Tokyo, Japan) for 20 min, and then total RNA was extracted and purified using TRIzol according to the manufacturer's protocol (Takara). Reverse transcription was performed using a ReverTra Ace qPCR RT-Kit (Takara). The resultant cDNAs were used as templates for RT-PCR with primers designed to detect the *algD*, *algR*, and *algU* genes, with parallel amplification of the *rpoD* gene as an internal control, all as previously described (Table 1). The primers used in this experiment were in accordance with another experiment [24]. RT-PCR was then performed with a Bio-Rad Real-Time PCR instrument using the SsoFast Evagreen Supermix Kit (Bio-Rad, CA, USA). The reaction procedure was as follows: 95°C for 30 s; 40 cycles of 95°C for 5 s and 60°C for 5 s; and a final melting curve from 65°C to 95°C, increasing by 0.5°C every 5 s. RT-PCR amplifications were conducted in triplicate. After RT-PCR amplification, the comparative threshold method (ΔΔCt analysis) was applied to evaluate the relative changes in gene expression in the RT-PCR experiments. The computer programs GenEx (Bio-Rad) and Excel (Microsoft) were used to solve the following equation: ΔΔCt = ΔCt sample − ΔCt reference (Bio-Rad) [27].

### 2.7. GDP-Mannose Dehydrogenase (GMD) Activity

Biofilms were made in the manner described above, with or without activated PMN or H_2_O_2_ treatments. The peg lids were incubated in a water bath sonicator (Tomy UD-201, Tokyo, Japan) for 20 min, and the bacteria suspension was then reincubated in LB at 37°C overnight, as described elsewhere [24, 28]. The cells were harvested by centrifugation (Tokyo, Japan) at 10,000*g* and 4°C for 20 min. The cell pellets were washed three times with PBS. The cells were then resuspended and disrupted in 2 ml of sonication buffer (pH = 7.0) containing 10 mM 3-(N-morpholino)propanesulfonic acid (MOPS), 0.5 mM phenylmethylsulfonyl fluoride (PMSF), and 2 mM dithiothreitol (DTT) with an ultrasonic disrupter for 20 min. The final supernatant (enzyme solution) was obtained by removing cell debris by centrifugation at 40,000*g* and 4°C for 40 min and then filtering the solution with a membrane filter (0.2 *μ*m). All operations were performed at 4°C.

For the placebo control group, which was the blank correction unit, 50 *μ*l of enzyme solution was added to 500 *μ*l of substrate solution (50 mM Tris HCl, 10 mM MgCl_2_, and 1 mM nicotinamide adenine dinucleotide (NAD)). To use glucose-6-phosphate dehydrogenase (G6PDH) as the enzyme control for determining enzyme activity, 50 *μ*l of enzyme solution was added to 500 *μ*l of substrate solution composed of 50 mM Tris–HCl, 10 mM MgCl_2_, 1 mM NAD, and 1 mM GDP-D-mannose at pH 7.5. To determine the GMD activity, 50 *μ*l of enzyme solution was added to 500 *μ*l of substrate solution (50 mM Tris–HCl, 5 mM MgCl_2_, 0.4 mM NAD phosphate, and 1 mM glucose-6-phosphate, pH 7.0). The enzymatic activities of GMD and G6PDH were assayed by determining the optical density (OD) at 340 nm at 37°C for 1 min using a spectrophotometer. The units of G6PDH and GMD activity in both groups are reported as ΔOD/mg protein. Total protein was assayed using the Coomassie brilliant blue method. Quantitation of protein was performed by measuring the absorbance at 595 nm. The quantity of protein that was present was determined using bovine serum albumin (BSA) as the assay standard.

### 2.8. Biofilm Staining and Confocal Laser Scanning Microscopy

*P. aeruginosa* FRD1 cells (OD600 = 0.1) were inoculated into Jensen's chemically defined media and added to a sterile 24-well plate containing glass coverslips (Costar, USA). The cultures were then incubated at 37°C without agitation. One or three days later, the biofilms were treated with PMNs (activated with 100 ng/ml PMA, 2 × 10^6^ cells/ml) and H_2_O_2_ (1 mM or 2 mM) and doubly distilled water for 120 min every time, three times per day [12], for one or two days. When the treatment ended, coverslips were rinsed three times with PBS and stained with SYTO 9 and propidium iodide from the LIVE/DEAD BacLight Bacterial Viability Kit according to the manufacturer's instructions (Invitrogen Molecular Probes, USA); the coverslips were then examined using confocal laser scanning microscopy (CLSM) [29].

Biofilms were stained with SYTO 9 and propidium iodide from the LIVE/DEAD kit for 15 min at room temperature in the dark and then washed three times with PBS. After the biofilms were stained, they were observed under a Nikon A1R laser confocal microscope (Nikon, Tokyo, Japan) with an argon laser at 488 nm (emission: 515 nm) and 543 nm (emission: 600 nm). Live bacteria were stained green, and dead bacteria were stained red. The thickness of the biofilms was calculated with the use of NIS-element AR4.5 (Nikon) [30, 31]. Biofilm staining repeated 2 times, and 3 replicates were in each group.

### 2.9. Statistical Analysis

Data are expressed as the mean ± standard deviation. The *t*-statistic was used to determine significant differences between two groups. One-way analysis of variance (ANOVA) was used for multigroup comparisons. Statistical analyses were performed using SPSS version 17.0 (SPSS Inc., Chicago, IL, USA). A *P* value less than 0.05 was considered to indicate the presence of a statistically significant difference. All experiments were repeated three times, and 2 replicates were in each group every time unless stated otherwise.

## 3. Results

### 3.1. Effect of PMNs or H_2_O_2_ on the Adhesion of Mucoid *P. aeruginosa* FRD1

We hypothesized that activated PMNs and their release of toxic oxygen by-products in CF lung environments could play a role in enhancing the biofilm formation of mucoid *P. aeruginosa* during the inflammatory response. As an attachment to an abiotic surface was the critical first step in biofilm formation, we examined the bacterial adhesion of *P. aeruginosa* FRD1. Considering that alginate interferes with the adhesion of mucoid *P. aeruginosa* [20] and the *P. aeruginosa* FRD1 in the experiment could not attach to the pegs well in the first several hours (data not shown), we tested the effects of activated PMNs and H_2_O_2_ on the adhesion of FRD1 during the first day of culture.

**Figure 1:**
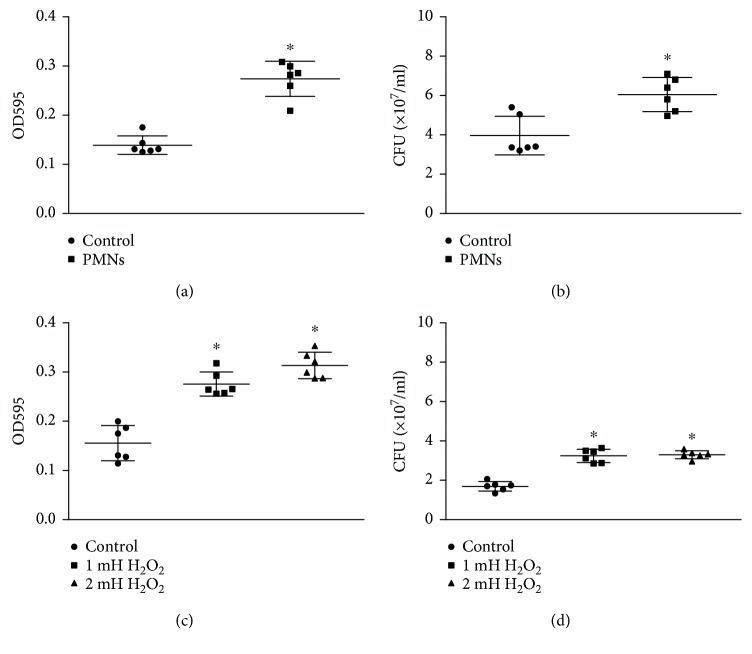
Effect of PMNs and H_2_O_2_ on the adhesion of mucoid *P. aeruginosa* FRD1. The error bars indicate standard deviations. Control: control group; PMNs: FRD1 group treated with PMNs; 1 mM H_2_O_2_: FRD1 group treated with 1 mM H_2_O_2_; 2 mM H_2_O_2_: FRD1 group treated with 2 mM H_2_O_2_. (a) Quantification of biofilm formation using crystal violet staining. Cells were grown in Jensen's medium for one day, in the presence of PMNs. PMNs increased the adhesion of *P. aeruginosa* FRD1. (b) The effect of PMNs on the number of viable cells is expressed in colony-forming units (CFUs). (c) Quantification of biofilm formation by crystal violet staining. H_2_O_2_ increased the adhesion of *P. aeruginosa* FRD1. (d) The effect of H_2_O_2_ on the number of viable cells is expressed in colony-forming units (CFUs). Data are presented as the mean ± SD (*n* = 6 in each treatment). ^∗^ *P* < 0.05 compared to the control group.

Bacterial adhesion in the groups treated with activated PMNs was significantly greater than that in the control groups (Figure 1(a)) (*P* < 0.05, Student's *t*-test), and the number of live *P. aeruginosa* that adhered to a polystyrene surface also significantly increased with activated PMN treatment (Figure 1(b)) (*P* < 0.05, Student's *t*-test). Because the production of ROS from PMNs is a critical mechanism during the immune response, we further tested the effects of H_2_O_2_ on the adhesion of *P. aeruginosa* FRD1: *P. aeruginosa* FRD1 was treated with sublethal concentrations of H_2_O_2_. Similar to the changes with PMN treatment, H_2_O_2_ significantly increased both adhesion (Figure 1(c)) (*P* < 0.05, one-way ANOVA) and the numbers of live *P. aeruginosa* FRD1 that adhered to the polystyrene surface (Figure 1(d)) (*P* < 0.05, one-way ANOVA).

### 3.2. Effect of PMNs or H_2_O_2_ on FRD1 Biofilms

**Figure 2:**
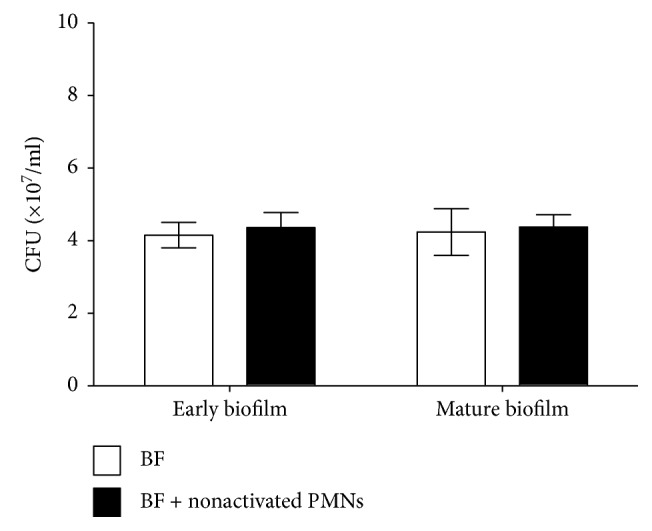
Effect of nonactivated PMNs on viable cells of biofilms of mucoid *P. aeruginosa* FRD1. The numbers of viable cells in biofilms treated with PMNs are expressed in colony-forming units (CFUs). The error bars indicate standard deviations. BF: biofilm without PMNs; BF + nonactivated PMNs: biofilm treated with nonactivated PMNs. Data are presented as the mean ± SD (*n* = 6 in each treatment).

**Figure 3:**
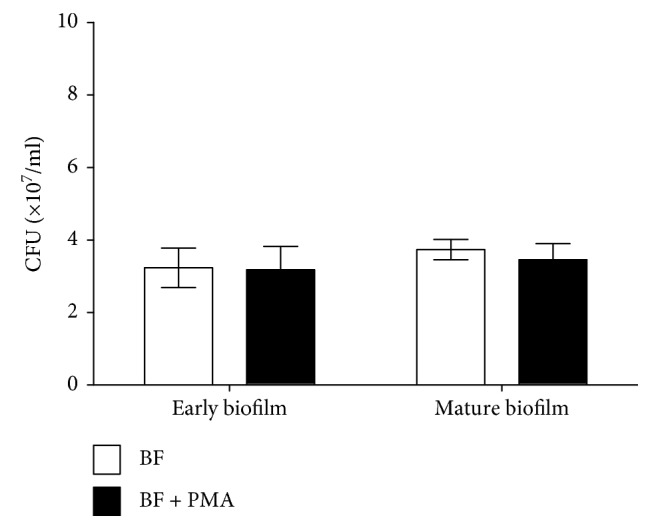
Effect of PMA on viable cells of biofilms of mucoid *P. aeruginosa* FRD1. The numbers of viable cells in biofilms treated with PMA are expressed in colony-forming units (CFUs). The error bars indicate standard deviations. BF: biofilm without PMA; BF + PMA: biofilm treated with PMA (100 ng/ml). Data are presented as the mean ± SD (*n* = 6 in each treatment).

To determine the effects of PMNs or H_2_O_2_ on the biofilms of FRD1, we examined the numbers of live *P. aeruginosa* in biofilms and the biofilm thickness using plate-counting assays and CLSM, respectively, after activated PMN or H_2_O_2_ treatment. PMA was used to stimulate PMNs before treatment, so we investigated the effect of nonactivated PMNs on biofilm, and the number of live *P. aeruginosa* FRD1 cells in biofilm did not differ significantly between the two groups (Figure 2). Then, we examined the viable cells in biofilm treated with PMA alone, and the viable cells in biofilm did not differ significantly between the two groups (Figure 3).

**Figure 4:**
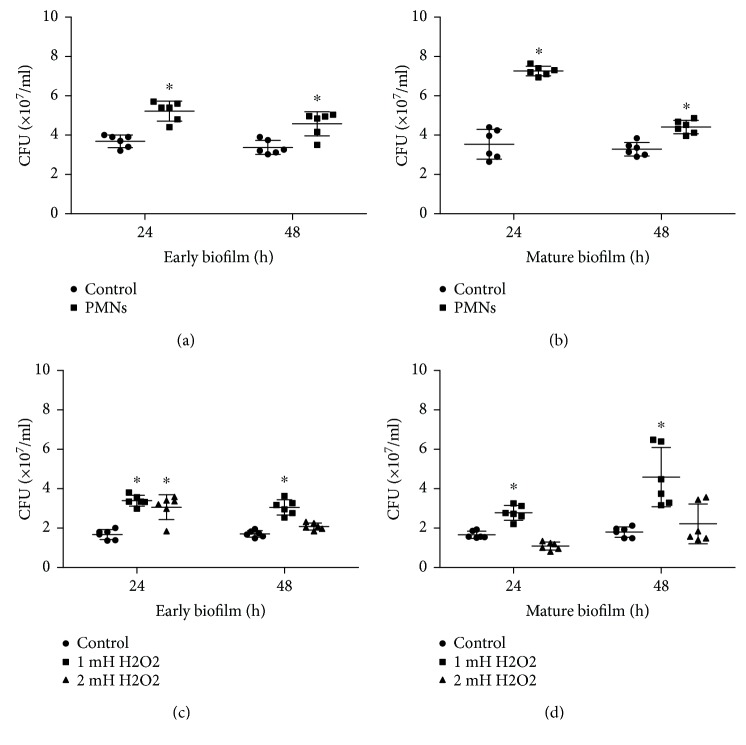
Effect of PMNs and H_2_O_2_ on viable cells of biofilms of mucoid *P. aeruginosa* FRD1. The error bars indicate standard deviations. Control: control group; PMNs: FRD1 biofilm group treated with PMNs; 1 mM H_2_O_2_: FRD1 biofilm group treated with 1 mM H_2_O_2_; 2 mM H_2_O_2_: FRD1 biofilm group treated with 2 mM H_2_O_2_. (a, b) The numbers of viable cells in biofilms treated with PMNs are expressed in colony-forming units (CFUs). (c, d) The numbers of viable cells in biofilms treated with H_2_O_2_ are expressed in colony-forming units (CFUs). Data are presented as the mean ± SD (*n* = 6 in each treatment). ^∗^ *P* < 0.05 compared to the control group.

**Figure 5:**
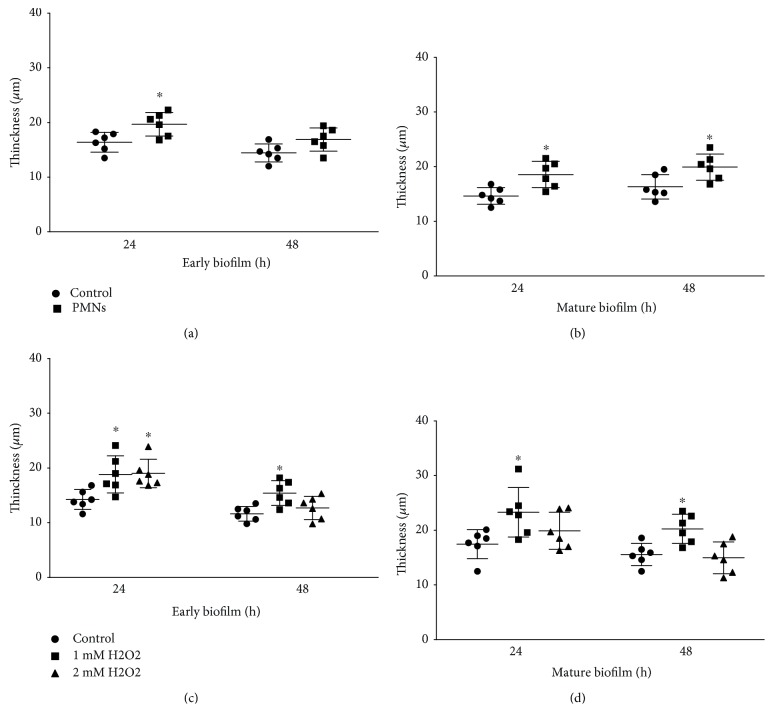
Effect of PMNs and H_2_O_2_ on thickness of biofilms of mucoid *P. aeruginosa* FRD1. The error bars indicate standard deviations. Control: control group; PMNs: FRD1 biofilm group treated with PMNs; 1 mM H_2_O_2_: FRD1 biofilm group treated with 1 mM H_2_O_2_; 2 mM H_2_O_2_: FRD1 biofilm group treated with 2 mM H_2_O_2_. (a, b) The thickness of biofilms treated with PMNs was determined using confocal laser-scanning microscopy. (c, d) The thickness of biofilms treated with H_2_O_2_ was determined using confocal laser-scanning microscopy. Data are presented as the mean ± SD (*n* = 6 in each treatment). ^∗^ *P* < 0.05 compared to the control group.

**Figure 6:**
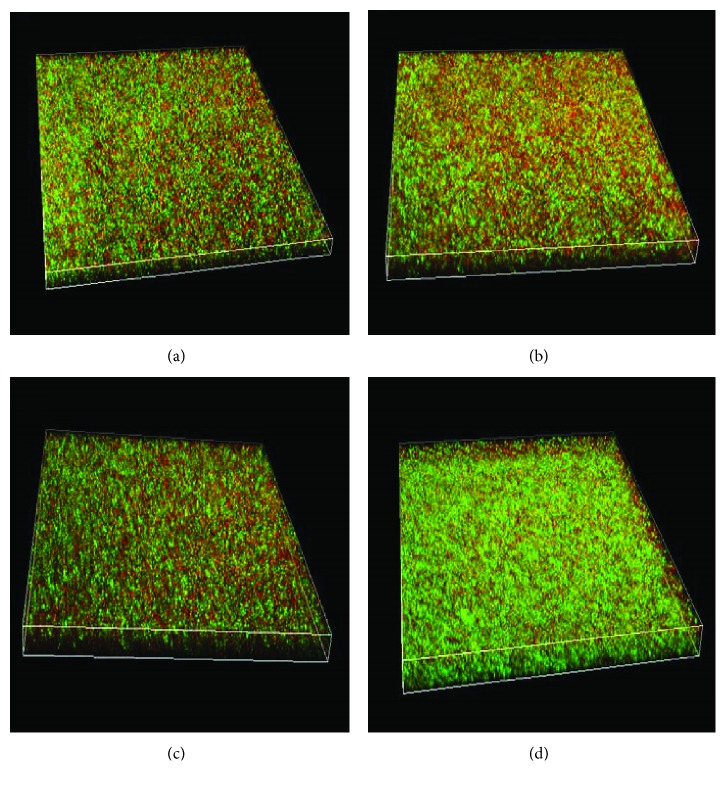
Effect of PMNs on biofilms of mucoid *P. aeruginosa* FRD1. Confocal laser scanning micrographs of *P. aeruginosa* FRD1 biofilms treated with PMNs. (a) Control group, early biofilm treated without PMNs. (b) Early biofilm treated with PMNs. (c) Mature biofilm treated without PMNs. (d) Mature biofilm treated with PMNs. Cells staining red are considered dead while cells staining green are viable cells.

The number of live *P. aeruginosa* FRD1 cells in early and mature biofilms was significantly increased after activated PMN treatment (Figures 4(a) and 4(b), *P* < 0.05, Student's *t*-test). In addition, the thickness of early biofilms increased significantly after a 24-hour treatment with activated PMNs (Figure 5(a), *P* < 0.05, Student's *t*-test), but not after a 48-hour treatment with activated PMN. The thickness of mature biofilms was increased by activated PMN treatments (Figure 5(b), *P* < 0.05, Student's *t*-test), which was consistent with the effect of activated PMNs on the three-dimensional structure of the biofilm (Figure 6) and the micrographs of biofilm (Figures S5–8, S11, S12, and S15).

Similarly, in early biofilms, the numbers of live *P. aeruginosa* cells were significantly increased after treatment with H_2_O_2_ (Figure 4(c), *P* < 0.05, one-way ANOVA). In mature biofilms, treatment with H_2_O_2_ increased the numbers of *P. aeruginosa* (Figure 4(d), *P* < 0.05, one-way ANOVA). Treatment with H_2_O_2_ significantly and consistently increased the thickness of early biofilms (Figure 5(c), *P* < 0.05, one-way ANOVA) and the thickness of mature biofilms (Figure 5(d), *P* < 0.05, one-way ANOVA). Micrographs of biofilm were according to the thickness of biofilm treated by H_2_O_2_ (Figures S1–4, S9, S10, S13, and S14).

### 3.3. Effect of PMNs or H_2_O_2_ on Alginate Contents

Because alginate is the main ingredient in the extracellular matrix of mucoid *P. aeruginosa* biofilms, we investigated the effects of PMNs or H_2_O_2_ on the alginate of mucoid *P. aeruginosa* FRD1.

**Figure 7:**
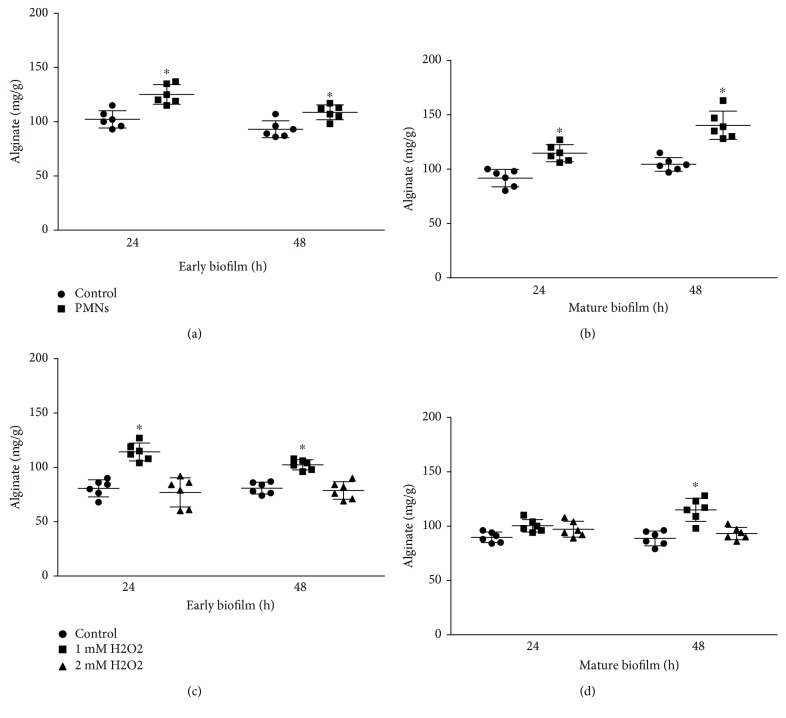
Effect of PMNs and H_2_O_2_ on alginate content. The error bars indicate standard deviations. Control: control group; PMNs: FRD1 biofilm group treated with PMNs; 1 mM H_2_O_2_: FRD1 biofilm group treated with 1 mM H_2_O_2_; 2 mM H_2_O_2_: FRD1 biofilm group treated with 2 mM H_2_O_2_. (a, b)The alginate content of *P. aeruginosa* FRD1 biofilms treated with PMNs. (c, d) The alginate content of *P. aeruginosa* FRD1 biofilms treated with H_2_O_2_. Data are presented as the mean ± SD (*n* = 6 in each treatment).^∗^ *P* < 0.05 compared to the control group.

In early and mature biofilms, the production of alginate of *P. aeruginosa* was significantly increased by treatment with activated PMNs (Figures 7(a) and 7(b), *P* < 0.05, Student's *t*-test). In early biofilms, treatment with H_2_O_2_ significantly increased alginate production (Figure 7(c), *P* < 0.05, one-way ANOVA). However, in mature biofilms, alginate production was increased only by treatment with H_2_O_2_ for 48 hours (Figure 7(d), *P* < 0.05, one-way ANOVA).

### 3.4. Effects of PMNs or H_2_O_2_ on the Expression of Genes Involved in Alginate Biosynthesis

**Figure 8:**
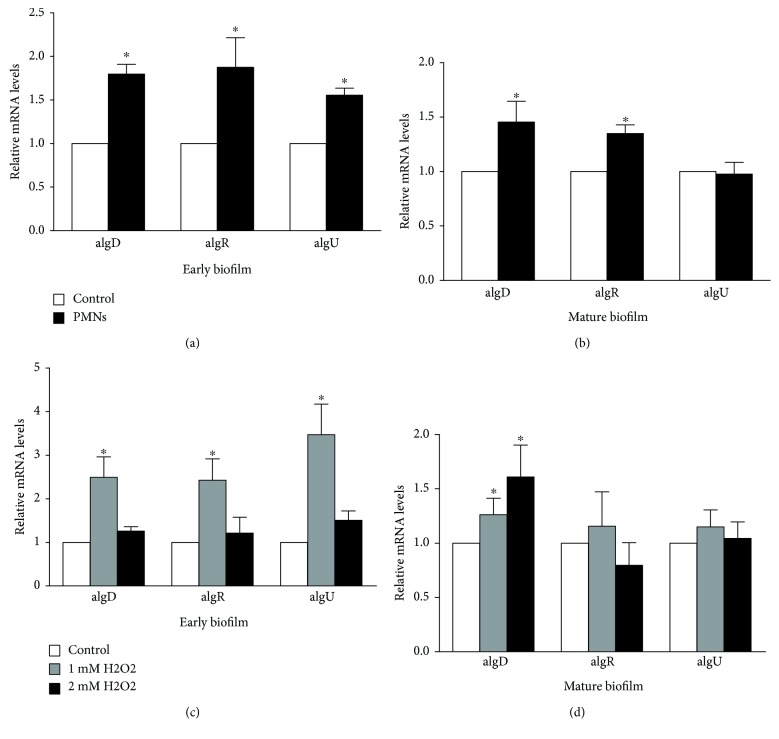
Effects of PMNs and H_2_O_2_ on the expression of genes involved in alginate biosynthesis. Control: control group; PMNs: FRD1 biofilm group treated with PMNs; 1 mM H_2_O_2_: FRD1 biofilm group treated with 1 mM H_2_O_2_; 2 mM H_2_O_2_: FRD1 biofilm group treated with 2 mM H_2_O_2_. Bacteria were grown in Jensen's medium. RNA was extracted from the bacteria, and the relative mRNA levels of *algD*, *algR*, and *algU* were determined by RT-PCR. (a, b) The expression of genes involved in alginate biosynthesis after treatment with PMNs. (c, d) The expression of genes involved in alginate biosynthesis after treatment with H_2_O_2_. Data are presented as the mean ± SD (*n* = 6 in each treatment). ^∗^ *P* < 0.05 compared to the control group.

We also used RT-PCR to evaluate the effects of PMNs or H_2_O_2_ on the expression of genes (*algD*, *algU*, and *algR*) that are involved in alginate biosynthesis in mucoid *P. aeruginosa* FRD1 biofilms. The expression of *algD*, *algU*, and *algR* in early biofilms was significantly upregulated by treatment with activated PMNs (Figure 8(a), *P* < 0.05, one-way ANOVA). In mature biofilms, only the expression of *algD* and *algR* genes was significantly upregulated by activated PMN treatment (Figure 8(b), *P* < 0.05, one-way ANOVA); the expression of *algU* was not influenced by PMN treatments.

In early biofilms, treatment with H_2_O_2_ significantly increased the expression of *algD*, *algU*, and *algR* (Figure 8(c), *P* < 0.05, one-way ANOVA). In mature biofilms, H_2_O_2_ treatment increased the expression of *algD* (Figure 8(d), *P* < 0.05, one-way ANOVA) but not the expression of *algU* and *algR*.

### 3.5. Effect of PMNs on GMD Activity

**Figure 9:**
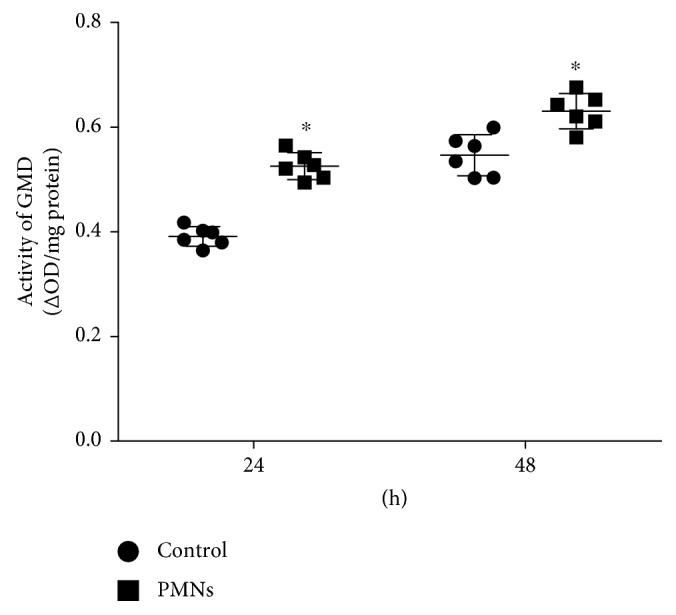
Effect of PMNs on GMD activity. GMD: GDP-mannose dehydrogenase; Control: *P. aeruginosa* FRD1 biofilm grown for 24 hours or 48 hours without PMNs; PMN: *P. aeruginosa* FRD1 biofilm treated with PMNs for 24 hours or 48 hours. Activities of GMD in FRD1 biofilms treated with PMNs were significantly different from those in FRD1 biofilms without PMNs. Data are presented as the means ± SD (*n* = 6 in each treatment). ^∗^ *P* < 0.05 compared to the control group.

**Figure 10:**
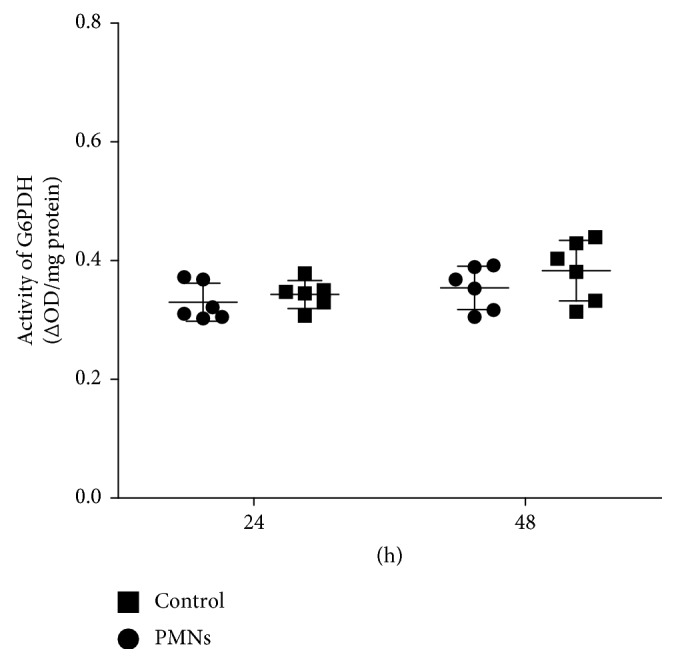
Effect of PMNs on G6PDH activity. G6PDH: glucose-6-phosphate dehydrogenase; Control: *P. aeruginosa* FRD1 biofilm grown for 24 hours or 48 hours without PMNs; PMN: *P. aeruginosa* FRD1 biofilm treated with PMNs for 24 hours or 48 hours. The activity of G6PDH did not differ significantly between the two groups. Data are presented as the mean ± SD (*n* = 6 in each treatment).

GMD is an enzyme that plays a key role in alginate biosynthesis. The effect of PMNs on GMD activity was determined using spectrophotometry. We compared the activity of glucose-6-phosphate dehydrogenase (G6PDH), which is present in the inner membrane of *Pseudomonas* and is related to glucose synthesis, to that of GMD [20]. The activity of GMD (ΔOD/mg protein) was increased significantly by activated PMN treatment (Figure 9, *P* < 0.05, Student's *t*-test), but the G6PDH activity was not affected by PMN treatment (Figure 10).

## 4. Discussion

*P. aeruginosa* biofilms are the main cause of persistent infection in patients with CF [2]; once present as a biofilm, *P. aeruginosa* has the ability to resist inflammatory conditions and antibiotic treatment for decades without eradication [13]. PMNs are the major host cell population in CF sputum; the host cell population in sputum consists of leucocytes and epithelial cells, and PMNs constitute 96 to 99% of the leucocytes [32]. PMNs are well known to be the first line of host defense against bacterial infection. However, when bacteria grow as biofilms, they are protected from the bactericidal activity of PMNs and thus cause persistent infections [33]. A previous study reported that PMN phenotype is usually different in CF patients. For example, in CF patients, TLR2, TLR4, TLR5, and TLR9 expression was increased on airway PMNs compared with circulating PMNs, and TLR5 activation enhanced the respiratory burst activity of PMNs [34], and the ROS output from PMNs of CF did not differ from that of controls [7]. However, Houston et al. reported that CF airway PMNs display functional exhaustion, and ROS production was reduced in sputum compared to blood PMNs [35]. So differences in PMNs function in sputum and blood likely relate to the different inflammatory milieu from which the cells were isolated. QS has been implicated in the differentiation, architecture, and virulence factors of *P. aeruginosa* biofilms [36]. Pacheco et al. demonstrated that the presence of PMNs could upregulate the synthesis of some QS-controlled virulence factors, including rhamnolipids, in wild-type *P. aeruginosa* [9]. These results were also supported by in vitro experiments by Alhede et al. that activated PMNs promoted the secretion of rhamnolipids, whereas inactivated PMNs did not increase rhamnolipid production [37]. We thus speculate that the oxygen by-products that are released from activated PMNs are essential for this process because ROS are important products when PMNs are challenged. An analysis of microarray data revealed that exposure of *P. aeruginosa* PAO1 to ROS substantially affected metabolism and bacterial virulence pathways [38]. Palma et al. also showed, using a transcriptome analysis, that H_2_O_2_ affected the expression of some genes related to PAO1 metabolism [39]. Therefore, ROS likely play an important role in the interaction between PMNs and *P. aeruginosa* biofilms.

*P. aeruginosa* strains isolated during the initial infection period are likely to be wild strains that are motile and nonmucoid [4]. After years of infection, strains of *P. aeruginosa* exhibit an extensive array of altered phenotypes, including a mucoid, nonmotile phenotype [40]. Mucoid *P. aeruginosa* exhibits enhanced microcolony formation [41], and colonization by mucoid *P. aeruginosa* is recognized as an indicator of a poor prognosis because these bacteria are seldom eradicated. Unfortunately, previous studies focused on the effect of activated PMNs or ROS on wild-type *P. aeruginosa* [11, 33, 37, 39, 42]. While *P. aeruginosa* biofilm formation in the CF airways appears to occur when mucous plugs develop, most strains isolated from the lungs of patients in the advanced stages of CF have a mucoid colony morphology [40, 43]. This morphology is usually accompanied by an overproduction of alginate, which makes it difficult to eradicate the *P. aeruginosa* infection using existing therapies [44]. Kragh et al. reported that PMNs restricted *P. aeruginosa* growth in biofilms *in vivo* and *in vitro*, because the activity of PMNs is the major cause of O_2_ depletion, rendering the *P. aeruginosa* aggregates anoxic to restrict the growth of biofilm [42]. Although PMNs play a key role in attacking and clearing *P. aeruginosa* in immunocompetent individuals, PMNs fail to do so in CF [8]. However, the strain of *P. aeruginosa* used in our experiment was mucoid *P. aeruginosa* FRD1, not the wild type, and FRD1 biofilms were grown using the previously described hanging-peg method with a small improvement that made the biofilm have sufficient oxygen. There was no study that has investigated the effect of PMNs or their active products on mucoid *P. aeruginosa* biofilms; we thus intended to determine whether they are effective against mucoid *P. aeruginosa* biofilms.

Although there are several types of ROS, including superoxide, hydrogen peroxide (H_2_O_2_), and the hydroxyl radical and these different species rapidly transition from one reactive oxygen intermediate to another, so some studies use sublethal concentrations of H_2_O_2_ to represent ROS *in vitro*, and the concentration of H_2_O_2_ is 1 mM or 2 mM [12, 39, 45]. Biofilm development involves at least three stages: initiation (adhesion), maturation, and detachment. In our study, we found that H_2_O_2_ or activated PMNs could significantly promote mucoid bacterial adhesion. It is seemingly impossible to eradicate bacteria once they form a biofilm, and mature biofilms are protected against the immune system and against antibiotics that are effective against planktonic *P. aeruginosa* [46]. In the present study, PMNs and H_2_O_2_ increased the thickness of the biofilm and the number of bacteria therein in the early and late phases of FRD1 biofilm maturation. Activated PMNs could thus promote the adhesion and maturation of biofilms, and ROS may also play an important role in these processes. FRD1, the strain that was used in these studies, mainly represents the strains that exist in the late phases of infections. The enhancement of biofilm formation that is described in this report occurred using a mucoid *P. aeruginosa*, so these results may represent a mechanism that allows mucoid *P. aeruginosa* to persist in CF airways.

To form biofilms, we attached bacteria to a surface, and they became embedded in a matrix of extracellular polymeric substances that they themselves produced. Alginate has been shown to be important for the formation of thick, highly structured biofilms, which contribute to clogging in CF lungs [20]. Increasing evidence has shown that persistent *P. aeruginosa* infections in patients with CF are composed of mucoid variants that produce alginate [47]. Alginate is the primary matrix component produced by mucoid *P. aeruginosa* and is a virulence factor that increases the resistance of biofilms to antibiotics and the immune system [48]. Hentzer et al. demonstrated that alginate decreased the sensitivity of biofilms to antibiotics and that a mucoid strain produced a biofilm in which the bacteria strongly resisted the antibiotic tobramycin [41]. The presence of alginate in biofilms also impedes the diffusion of some antimicrobial agents, thus protecting the bacteria from common bactericides [49]. Previous studies reported that alginate impaired the responses of CF airway epithelial cells and alveolar macrophages during inflammatory responses [50] and that bacteria encased in alginate were also protected from host defense mechanisms such as macrophage killing [51] and antibody-independent opsonic killing [52]. So activated PMNs could not kill FRD1 embedded with alginate in our study; on the contrary, it promoted the biofilm of FRD1. Furthermore, alginate can also impede macrophages and neutrophils that would normally kill pathogens [53, 54]. In the present study, we found that PMNs could enhance alginate production in FRD1 biofilms. Furthermore, Mathee et al. showed that the production of ROS from stimulated PMNs played an important role in the generation of mucoid variants during inflammatory responses to *P. aeruginosa* PAO1 [12]. In our study, H_2_O_2_ could also promote the production of alginate in FRD1 biofilms. After three days of culture, the biofilm goes into the maturation of microcolonies which are thick, three-dimensional structures encased in an extracellular matrix. Bacteria embedded within an extracellular matrix in mature biofilms exhibit greater resistance to stimulating, which may be the reason that H_2_O_2_ did not increase the content of alginate in a mature biofilm after 24 hours treatment; however, it promotes alginate content 48 hours later. As discussed above, PMNs and their active products may assist in these processes during persistent infections of mucoid strains because PMNs were consistently recruited to the infection site.

The mechanism for the expression of alginate is complex and requires several regulatory proteins that act in a hierarchical regulatory cascade [55]. Mucoid *P. aeruginosa* often possesses mutations in *mucA*, which is an anti-*σ* factor that controls the activity of *σ* ^22^ [56]. The top of the regulatory hierarchy is mediated by *σ* ^22^, which is encoded by *algU* [57–59]. Mutations in this negative regulator of *σ* ^22^ (*mucA*) increased the expression of *algU* by an autoregulatory mechanism [59] and increased the expression of the alginate biosynthetic operon, which is controlled by the *algD* promoter [60]. *AlgR* is the response regulator component of a two-component regulator that binds to three sites in the *algD* promoter [55]. The gene *algD*, which encodes GDP-mannose dehydrogenase, is the key gene in the biosynthetic operon [61]. This study showed that PMNs can affect alginate production by upregulating genes. The mechanisms involved in alginate biosynthesis have attracted many studies over the past few decades, but they are not fully understood. Therefore, the mechanism by which PMN administration changed gene expression in mucoid *P. aeruginosa* biofilms cannot be comprehensively explained.

Since genes related to alginate biosynthesis are being regulated, some enzymes, among which GMD is most crucial, may play important roles [24]. GMD is the most important enzyme in alginate synthesis because it functions during the last stage of alginate synthesis and has higher activity than other enzymes [62]. Accordingly, we observed that treatment with PMNs increased the activity of GMD without decreasing the activity of G6PDH. Gene transcription is therefore probably not the only manner in which PMNs influence alginate production. PMNs may influence alginate production by interfering with its control system at the gene expression and enzymatic levels.

Activated PMNs and the ROS that they release have also been shown to convert wild-type *P. aeruginosa* to its intractable mucoid form found in the CF lung [12]. Our findings further demonstrated that persistently recruiting PMNs promotes biofilm formation by mucoid *P. aeruginosa.* H_2_O_2_, the main ROS formed during oxidative bursts, can also enhance the development of mucoid *P. aeruginosa* biofilms instead of removing them. This mechanism may be important in permanently maintaining *P. aeruginosa* biofilms in CF patients.

## 5. Conclusions

It has been demonstrated that PMNs or H_2_O_2_ can promote the biofilm of mucoid *P. aeruginosa* FRD1 accompanied with the increase of alginate production. Suppressing the excessive oxidative respiratory burst of PMNs may be a promising approach to accelerate to eliminate biofilm infections by mucoid *P. aeruginosa*. It may thus be possible to use antioxidants and anti-inflammatory agents as preventive and therapeutic measures in CF patients with *P. aeruginosa* infections. However, we were unable to identify the molecular mechanisms by which PMNs or H_2_O_2_ regulate mucoid *P. aeruginosa*, which requires further immunological and biochemical studies.

## Figures and Tables

**Table 1:** Primers for genes amplified by RT-PCR.

Gene	Primer sequence
*algD-F*	5-GCTCAACCTGTCGCGCTACT-3
*algD-R*	5-GAACTCGCCACCACTTCGTC-3
*algU-F*	5-GATTGATCGTGCGGTTCGTG-3
*algU-R*	5-AAGATCCGCGACCGTACCGT-3
*algR -F*	5-GAAGAAGCGCTGACGCTGAT-3
*algR-R*	5-ATGGCGCAAGGTCACGTACT-3
*rpoD -F*	5-GGTGTGGTCGGTGTTCATGT-3
*rpoD-R*	5-CCGCAAGGTACTGAAGATCG-3

## Data Availability

The data used to support the findings of this study are included in the article and the supplementary materials.

## Supplementary Materials

Supplementary Materials Confocal laser scanning micrograph of *P. aeruginosa* FRD1 biofilms treated with PMNs or H_2_O_2_.

## References

[1] Lyczak J. B., Cannon C. L., Pier G. B. (2000). Establishment of *Pseudomonas aeruginosa* infection: lessons from a versatile opportunist. *Microbes and Infection*.

[2] Crull M. R., Ramos K. J., Caldwell E., Mayer-Hamblett N., Aitken M. L., Goss C. H. (2016). Change in Pseudomonas *aeruginosa* prevalence in cystic fibrosis adults over time. *BMC Pulmonary Medicine*.

[3] Spencer D. H., Kas A., Smith E. E. (2003). Whole-genome sequence variation among multiple isolates of *Pseudomonas aeruginosa*. *Journal of Bacteriology*.

[4] Folkesson A., Jelsbak L., Yang L. (2012). Adaptation of *Pseudomonas aeruginosa* to the cystic fibrosis airway: an evolutionary perspective. *Nature Reviews Microbiology*.

[5] Rasamiravaka T., Labtani Q., Duez P., El Jaziri M. (2015). The formation of biofilms by *Pseudomonas aeruginosa*: a review of the natural and synthetic compounds interfering with control mechanisms. *BioMed Research International*.

[6] Hoiby N., Bjarnsholt T., Givskov M., Molin S., Ciofu O. (2010). Antibiotic resistance of bacterial biofilms. *International Journal of Antimicrobial Agents*.

[7] McKeon D. J., Cadwallader K. A., Idris S. (2010). Cystic fibrosis neutrophils have normal intrinsic reactive oxygen species generation. *European Respiratory Journal*.

[8] Rada B. (2017). Interactions between neutrophils and Pseudomonas aeruginosa in cystic fibrosis. *Pathogens*.

[9] Pacheco G. J., Reis R. S., Fernandes A. C. L. B. (2012). Rhamnolipid production: effect of oxidative stress on virulence factors and proteome of *Pseudomonas aeruginosa* PA1. *Applied Microbiology and Biotechnology*.

[10] Kharazmi A., Bibi Z., Nielsen H., Hoiby N., Doring G. (1989). Effect of *Pseudomonas aeruginosa* rhamnolipid on human neutrophil and monocyte function. *APMIS*.

[11] Walker T. S., Tomlin K. L., Worthen G. S. (2005). Enhanced *Pseudomonas aeruginosa* biofilm development mediated by human neutrophils. *Infection and Immunity*.

[12] Mathee K., Ciofu O., Sternberg C. (1999). Mucoid conversion of Pseudomonas aeruginos by hydrogen peroxide: a mechanism for virulence activation in the cystic fibrosis lung. *Microbiology*.

[13] Jensen P. O., Kolpen M., Kragh K. N., Kuhl M. (2017). Microenvironmental characteristics and physiology of biofilms in chronic infections of CF patients are strongly affected by the host immune response. *APMIS*.

[14] Simpson J. A., Smith S. E., Dean R. T. (1989). Scavenging by alginate of free radicals released by macrophages. *Free Radical Biology and Medicine*.

[15] Ma L., Wang S., Wang D., Parsek M. R., Wozniak D. J. (2012). The roles of biofilm matrix polysaccharide Psl in mucoid *Pseudomonas aeruginosa* biofilms. *FEMS Immunology & Medical Microbiology*.

[16] Haslett C. H., Guthrie L. A., Kopaniak M. M., Johnston Jr R. B., Henson P. M. (1985). Modulation of multiple neutrophil functions by preparative methods or trace concentrations of bacterial lipopolysaccharide. *The American Journal of Pathology*.

[17] Ceri H., Olson M. E., Stremick C., Read R. R., Morck D., Buret A. (1999). The Calgary biofilm device: new technology for rapid determination of antibiotic susceptibilities of bacterial biofilms. *Journal of Clinical Microbiology*.

[18] Muhlebach M. S., Stewart P. W., Leigh M. W., Noah T. L. (1999). Quantitation of inflammatory responses to bacteria in young cystic fibrosis and control patients. *American Journal of Respiratory and Critical Care Medicine*.

[19] Muhlebach M. S., Noah T. L. (2002). Endotoxin activity and inflammatory markers in the airways of young patients with cystic fibrosis. *American Journal of Respiratory and Critical Care Medicine*.

[20] Hay I. D., Gatland K., Campisano A., Jordens J. Z., Rehm B. H. (2009). Impact of alginate overproduction on attachment and biofilm architecture of a supermucoid *Pseudomonas aeruginosa* strain. *Applied and Environmental Microbiology*.

[21] Ma S., Selvaraj U., Ohman D. E., Quarless R., Hassett D. J., Wozniak D. J. (1998). Phosphorylation-independent activity of the response regulators AlgB and AlgR in promoting alginate biosynthesis in mucoid *Pseudomonas aeruginosa*. *Journal of Bacteriology*.

[22] Jain S., Ohman D. E. (2005). Role of an alginate lyase for alginate transport in mucoid *Pseudomonas aeruginosa*. *Infection and Immunity*.

[23] Franklin M. J., Chitnis C. E., Gacesa P., Sonesson A., White D. C., Ohman D. E. (1994). Pseudomonas aeruginosa AlgG is a polymer level alginate C5-mannuronan epimerase. *Journal of Bacteriology*.

[24] Li F., Yu J., Yang H., Wan Z., Bai D. (2008). Effects of ambroxol on alginate of mature *Pseudomonas aeruginosa* biofilms. *Current Microbiology*.

[25] Knutson C. A., Jeanes A. (1968). A new modification of the carbazole analysis: application to heteropolysaccharides. *Analytical Biochemistry*.

[26] Pedersen S. S., Kharazmi A. R., Espersen F. R., Høiby N. (1990). Pseudomonas aeruginosa alginate in cystic fibrosis sputum and the inflammatory response. *Infection and Immunity*.

[27] Wang Z., Xiang Q., Yang T. (2016). Autoinducer-2 of *Streptococcus mitis* as a target molecule to inhibit pathogenic multi-species biofilm formation *in vitro* and in an endotracheal intubation rat model. *Frontiers in Microbiology*.

[28] Terry J. M., Pina S. E., Mattingly S. J. (1991). Environmental conditions which influence mucoid conversion Pseudomonas aeruginosa PAO1. *Infection and Immunity*.

[29] Strathmann M., Wingender J., Flemming H.-C. (2002). Application of fluorescently labelled lectins for the visualization and biochemical characterization of polysaccharides in biofilms of *Pseudomonas aeruginosa*. *Journal of Microbiological Methods*.

[30] Song S., Du L., Yu J. (2015). Does *Streptococcus mitis*, a neonatal oropharyngeal bacterium, influence the pathogenicity of *Pseudomonas aeruginosa*?. *Microbes and Infection*.

[31] Li H., Li X., Wang Z. (2015). Autoinducer-2 regulates *Pseudomonas aeruginosa* PAO1 biofilm formation and virulence production in a dose-dependent manner. *BMC Microbiology*.

[32] Hector A., Jonas F., Kappler M., Feilcke M., Hartl D., Griese M. (2010). Novel method to process cystic fibrosis sputum for determination of oxidative state. *Respiration*.

[33] van Gennip M., Christensen L. D., Alhede M. (2012). Interactions between polymorphonuclear leukocytes and *Pseudomonas aeruginosa* biofilms on silicone implants *in vivo*. *Infection and Immunity*.

[34] Koller B., Kappler M., Latzin P. (2008). TLR expression on neutrophils at the pulmonary site of infection: TLR1/TLR2-mediated up-regulation of TLR5 expression in cystic fibrosis lung disease. *The Journal of Immunology*.

[35] Houston N., Stewart N., Smith D. S., Bell S. C., Champion A. C., Reid D. W. (2012). Sputum neutrophils in cystic fibrosis patients display a reduced respiratory burst. *Journal of cystic fibrosis*.

[36] Davies D. G. (1998). The involvement of cell-to-cell signals in the development of a bacterial biofilm. *Science*.

[37] Alhede M., Bjarnsholt T., Jensen P. O. (2009). *Pseudomonas aeruginosa* recognizes and responds aggressively to the presence of polymorphonuclear leukocytes. *Microbiology*.

[38] Salunkhe P., Topfer T., Buer J., Tummler B. (2005). Genome-wide transcriptional profiling of the steady-state response of *Pseudomonas aeruginosa* to hydrogen peroxide. *Journal of Bacteriology*.

[39] Palma M., DeLuca D., Worgall S., Quadri L. E. N. (2003). Transcriptome analysis of the response of *Pseudomonas aeruginosa* to hydrogen peroxide. *Journal of Bacteriology*.

[40] Rac H., Stover K. R., Wagner J. L., King S. T., Warnock H. D., Barber K. E. (2017). Time–kill analysis of ceftolozane/tazobactam efficacy against mucoid *Pseudomonas aeruginosa* strains from cystic fibrosis patients. *Infectious diseases and therapy*.

[41] Hentzer M., Teitzel G. M., Balzer G. J. (2001). Alginate overproduction affects *Pseudomonas aeruginosa* biofilm structure and function. *Journal of Bacteriology*.

[42] Kragh K. N., Alhede M., Jensen P. Ø. (2014). Polymorphonuclear leukocytes restrict growth of *Pseudomonas aeruginosa* in the lungs of cystic fibrosis patients. *Infection and Immunity*.

[43] Worlitzsch D., Tarran R., Ulrich M. (2002). Effects of reduced mucus oxygen concentration in airway *Pseudomonas* infections of cystic fibrosis patients. *The Journal of Clinical Investigation*.

[44] Ghadam P., Akhlaghi F., Ali A. A. (2017). One-step purification and characterization of alginate lyase from a clinical Pseudomonas aeruginosa with destructive activity on bacterial biofilm. *Iranian journal of basic medical sciences*.

[45] Nakamura H. (1994). Adult T cell leukemia-derived factor/human thioredoxin protects endothelial F-2 cell injury caused by activated neutrophils or hydrogen peroxide. *Immunology Letters*.

[46] Jensen P. O., Briales A., Brochmann R. P. (2014). Formation of hydroxyl radicals contributes to the bactericidal activity of ciprofloxacin against *Pseudomonas aeruginosa* biofilms. *Pathogens and disease*.

[47] Lyczak J. B., Cannon C. L., Pier G. B. (2002). Lung infections associated with cystic fibrosis. *Clinical Microbiology Reviews*.

[48] Goltermann L., Tolker-Nielsen T. (2017). Importance of the exopolysaccharide matrix in antimicrobial tolerance of *Pseudomonas aeruginosa* aggregates. *Antimicrobial Agents and Chemotherapy*.

[49] Nivens D. E., Ohman D. E., Williams J., Franklin M. J. (2001). Role of alginate and its O acetylation in formation of *Pseudomonas aeruginosa* microcolonies and biofilms. *Journal of Bacteriology*.

[50] Chattoraj S. S., Murthy R., Ganesan S. (2010). *Pseudomonas aeruginosa* alginate promotes Burkholderia cenocepacia persistence in cystic fibrosis transmembrane conductance regulator knockout mice. *Infection and Immunity*.

[51] Leid J. G., Willson C. J., Shirtliff M. E., Hassett D. J., Parsek M. R., Jeffers A. K. (2005). The exopolysaccharide alginate protects *Pseudomonas aeruginosa* biofilm bacteria from IFN-*γ*-mediated macrophage killing. *The Journal of Immunology*.

[52] Pier G. B., Coleman F., Grout M., Franklin M., Ohman D. E. (2001). Role of alginate O acetylation in resistance of mucoid *Pseudomonas aeruginosa* to opsonic phagocytosis. *Infection and Immunity*.

[53] Simpson J. A., Smith S. E., Dean R. T. (1988). Alginate inhibition of the uptake of *Pseudomonas aeruginosa* by macrophages. *Microbiology*.

[54] Learn D. B., Brestel E. P., Seetharama S. U. (1987). Hypochlorite scavenging by Pseudomonas aeruginosa alginate. *Infection and Immunity*.

[55] Hay I. D., Ur Rehman Z., Ghafoor A., Rehm B. H. A. (2010). Bacterial biosynthesis of alginates. *Journal of Chemical Technology and Biotechnology*.

[56] Martin D. W., Schurr M. J., Mudd M. H., Govan J. R., Holloway B. W., Deretic V. (1993). Mechanism of conversion to mucoidy in Pseudomonas aeruginosa infecting cystic fibrosis patients. *Proceedings of the National Academy of Sciences of the United States of America*.

[57] Hershberger C. D., Ye R. W., Parsek M. R., Xie Z. D., Chakrabarty A. M. (1995). The algT (algU) gene of Pseudomonas aeruginosa, a key regulator involved in alginate biosynthesis, encodes an alternative sigma factor (sigma E). *Proceedings of the National Academy of Sciences of the United States of America*.

[58] Martin D. W., Schurr M. J., Yu H., Deretic V. (1994). Analysis of promoters controlled by the putative sigma factor AlgU regulating conversion to mucoidy in Pseudomonas aeruginosa: relationship to sigma E and stress response. *Journal of Bacteriology*.

[59] DeVries C. A., Ohman D. E. (1994). Mucoid-to-nonmucoid conversion in alginate-producing Pseudomonas aeruginosa often results from spontaneous mutations in algT, encoding a putative alternate sigma factor, and shows evidence for autoregulation. *Journal of Bacteriology*.

[60] Deretic V., Gill J. F., Chakrabarty A. M. (1987). Gene algD coding for GDPmannose dehydrogenase is transcriptionally activated in mucoid Pseudomonas aeruginosa. *Journal of Bacteriology*.

[61] Xu B., Soukup R. J., Jones C. J., Fishel R., Wozniak D. J. (2016). *Pseudomonas aeruginosa* AmrZ binds to four sites in the algD promoter, inducing DNA-AmrZ complex formation and transcriptional activation. *Journal of Bacteriology*.

[62] Tenhaken R., Voglas E., Cock J. M., Neu V., Huber C. G. (2011). Characterization of GDP-mannose dehydrogenase from the brown alga *Ectocarpus siliculosus* providing the precursor for the alginate polymer. *Journal of Biological Chemistry*.

