# Supplementary material for: Polymorphonuclear Leukocytes or Hydrogen Peroxide Enhance Biofilm Development of Mucoid Pseudomonas aeruginosa
Source: Mediators Inflamm. 2018 Jul 4;2018:8151362. doi: 10.1155/2018/8151362 (PMC6079396; doi:10.1155/2018/8151362)
Supplement: Supplementary Materials — Confocal laser scanning micrograph of P. aeruginosa FRD1 biofilms treated with PMNs or H2O2. [file 8151362.f1.pdf]

Supplementary Figure Legends

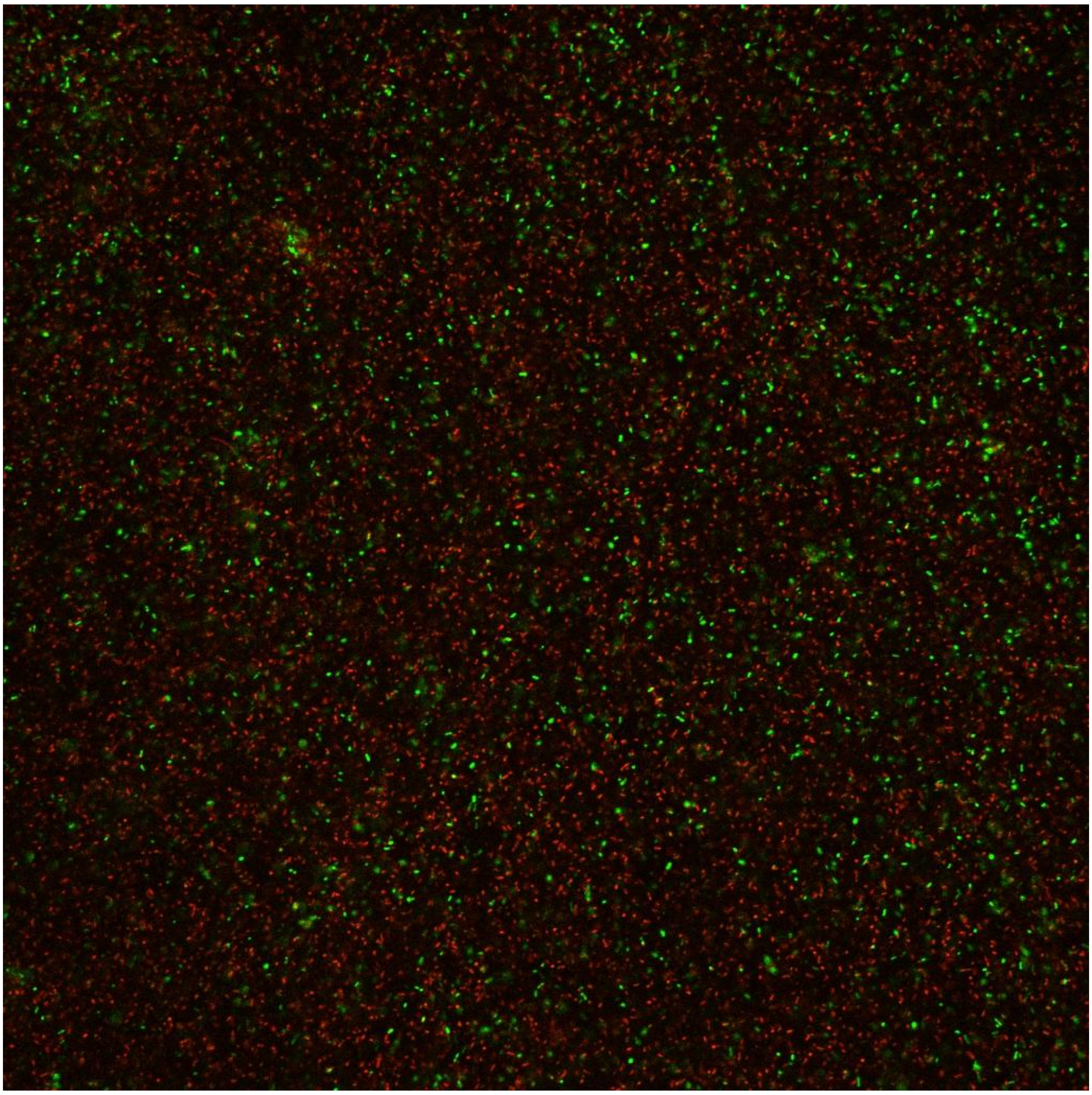

Fig. S1. Confocal laser scanning micrograph of early *P. aeruginosa* FRD1 biofilms treated with ddH$_2$O for 24 hours. Biofilms were stained with SYTO 9 and propidium iodide from the LIVE/DEAD kit and observed under a Nikon A1R laser confocal microscope (Nikon, Tokyo, Japan). Cells staining red are considered dead while cells staining green are viable cells.

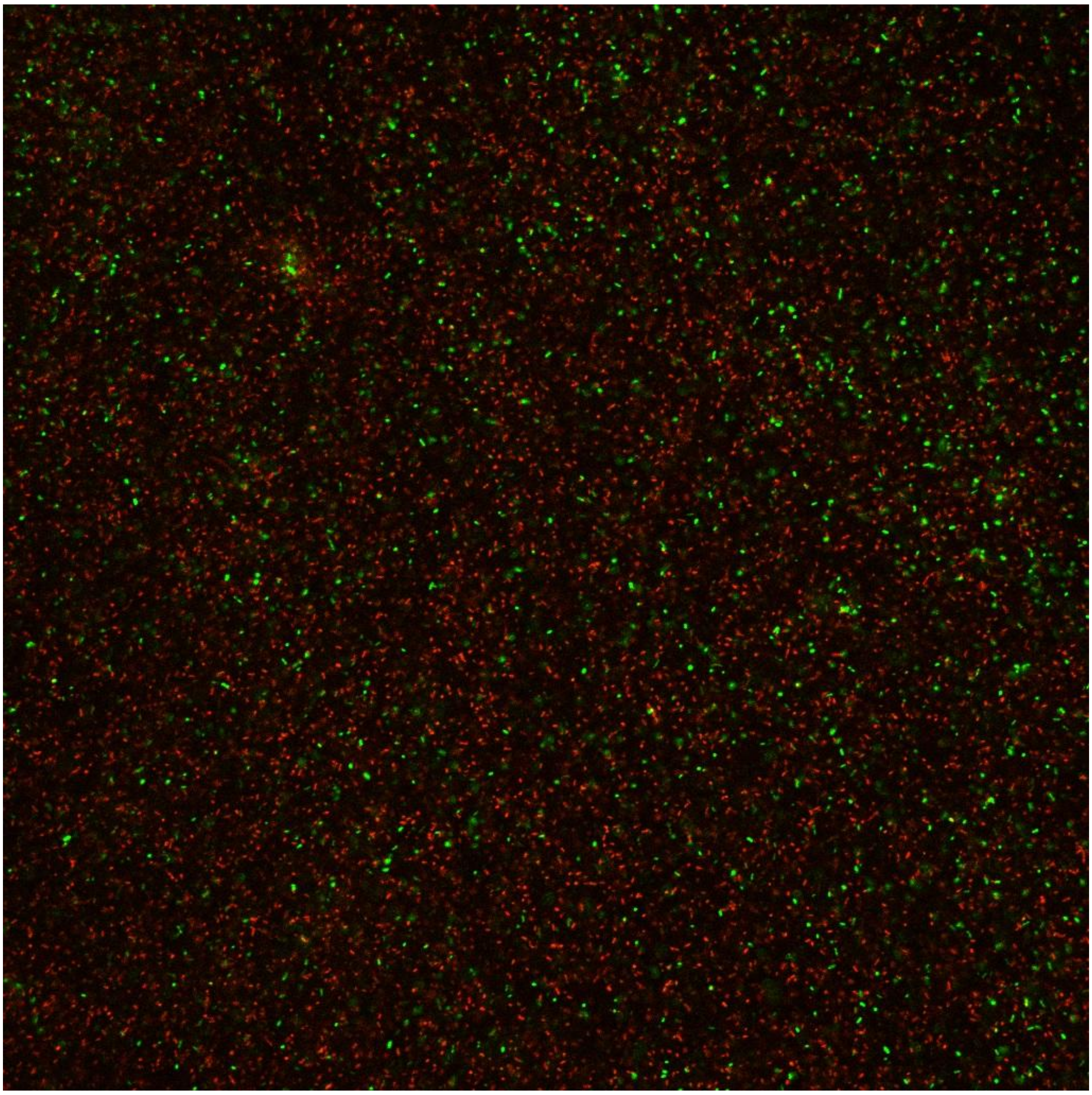

Fig. S2. Confocal laser scanning micrograph of early *P. aeruginosa* FRD1 biofilms treated with ddH$_2$O for 48 hours. Biofilms were stained with SYTO 9 and propidium iodide from the LIVE/DEAD kit and observed under a Nikon A1R laser confocal microscope (Nikon, Tokyo, Japan). Cells staining red are considered dead while cells staining green are viable cells.

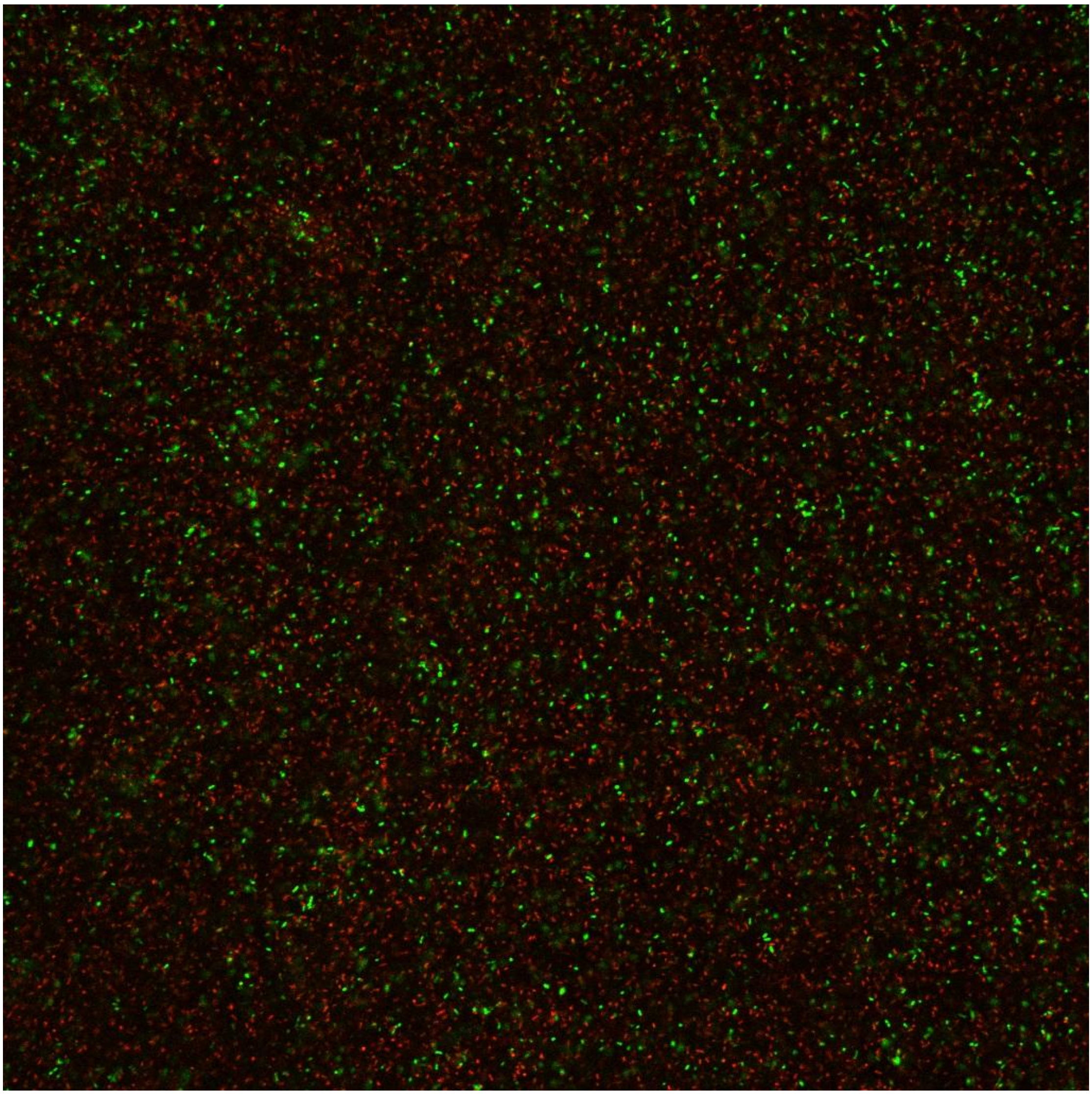

Fig. S3. Confocal laser scanning micrograph of mature *P. aeruginosa* FRD1 biofilms treated with ddH$_2$O for 24 hours. Biofilms were stained with SYTO 9 and propidium iodide from the LIVE/DEAD kit and observed under a Nikon A1R laser confocal microscope (Nikon, Tokyo, Japan). Cells staining red are considered dead while cells staining green are viable cells.

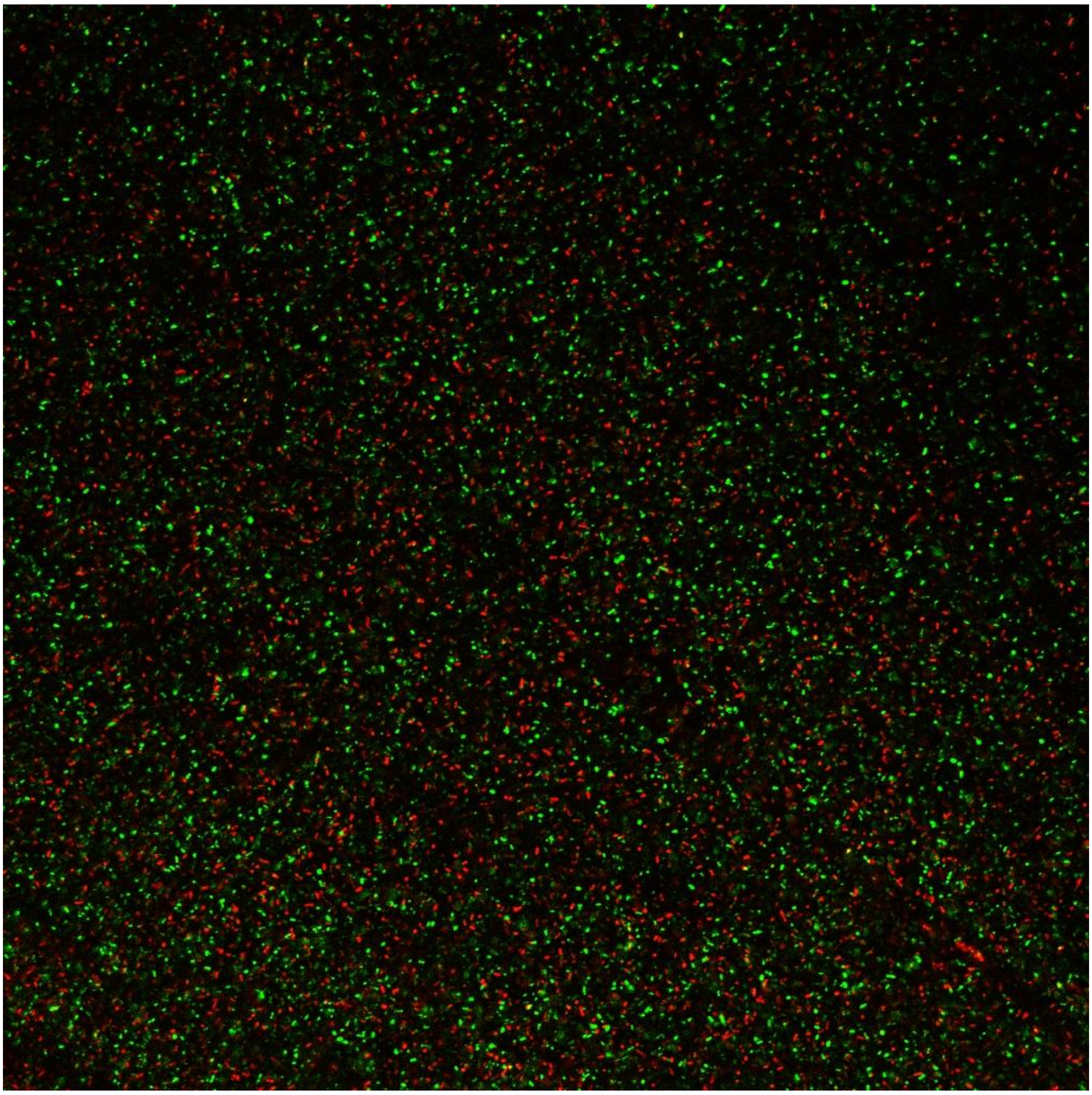

Fig. S4. Confocal laser scanning micrograph of mature *P. aeruginosa* FRD1 biofilms treated with ddH_2O for 48 hours. Biofilms were stained with SYTO 9 and propidium iodide from the LIVE/DEAD kit and observed under a Nikon A1R laser confocal microscope (Nikon, Tokyo, Japan). Cells staining red are considered dead while cells staining green are viable cells.

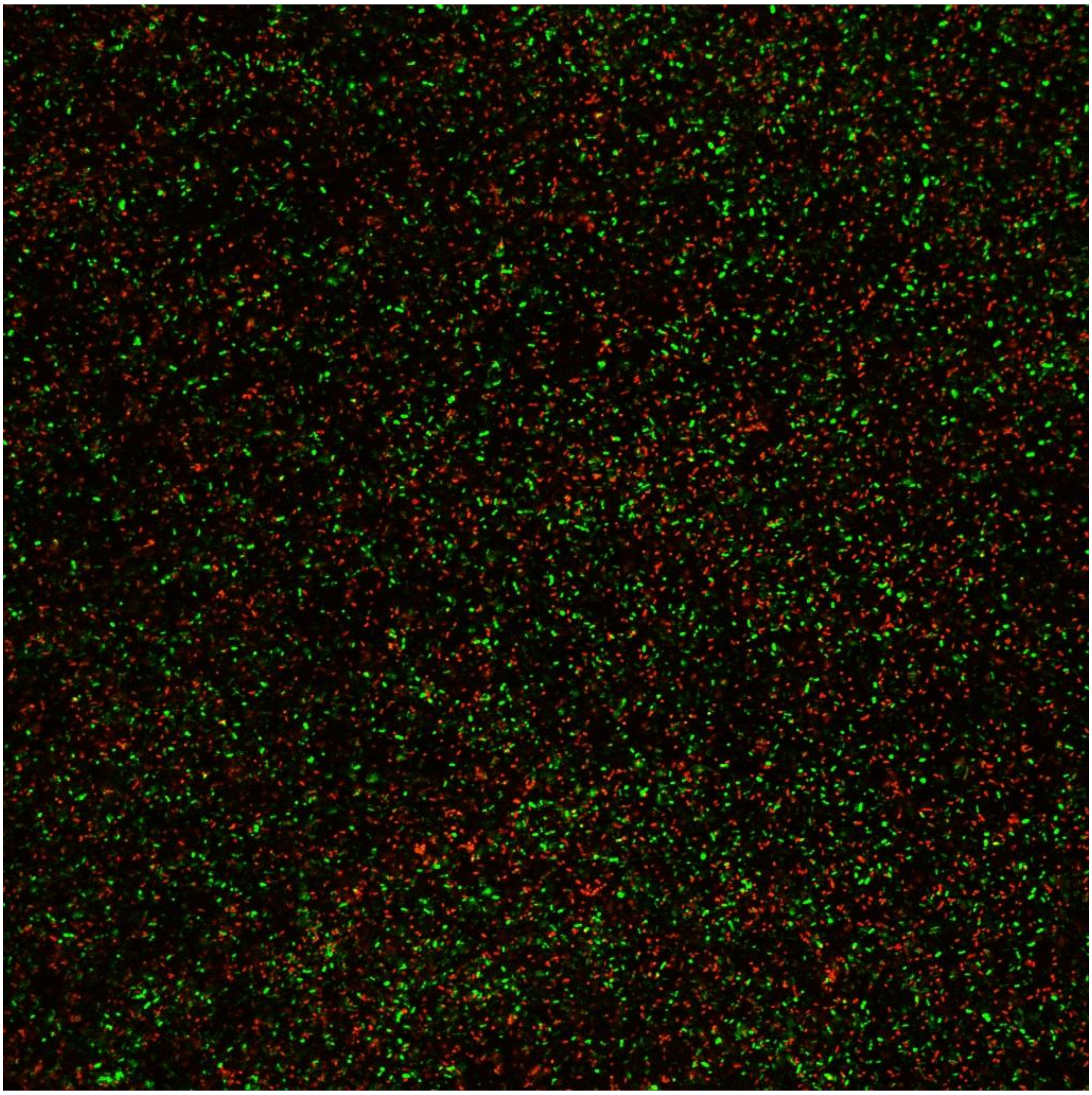

Fig. S5. Confocal laser scanning micrograph of early *P. aeruginosa* FRD1 biofilms treated with RPMI 1640 (without PMNs) for 24 hours. Biofilms were stained with SYTO 9 and propidium iodide from the LIVE/DEAD kit and observed under a Nikon A1R laser confocal microscope (Nikon, Tokyo, Japan). Cells staining red are considered dead while cells staining green are viable cells.

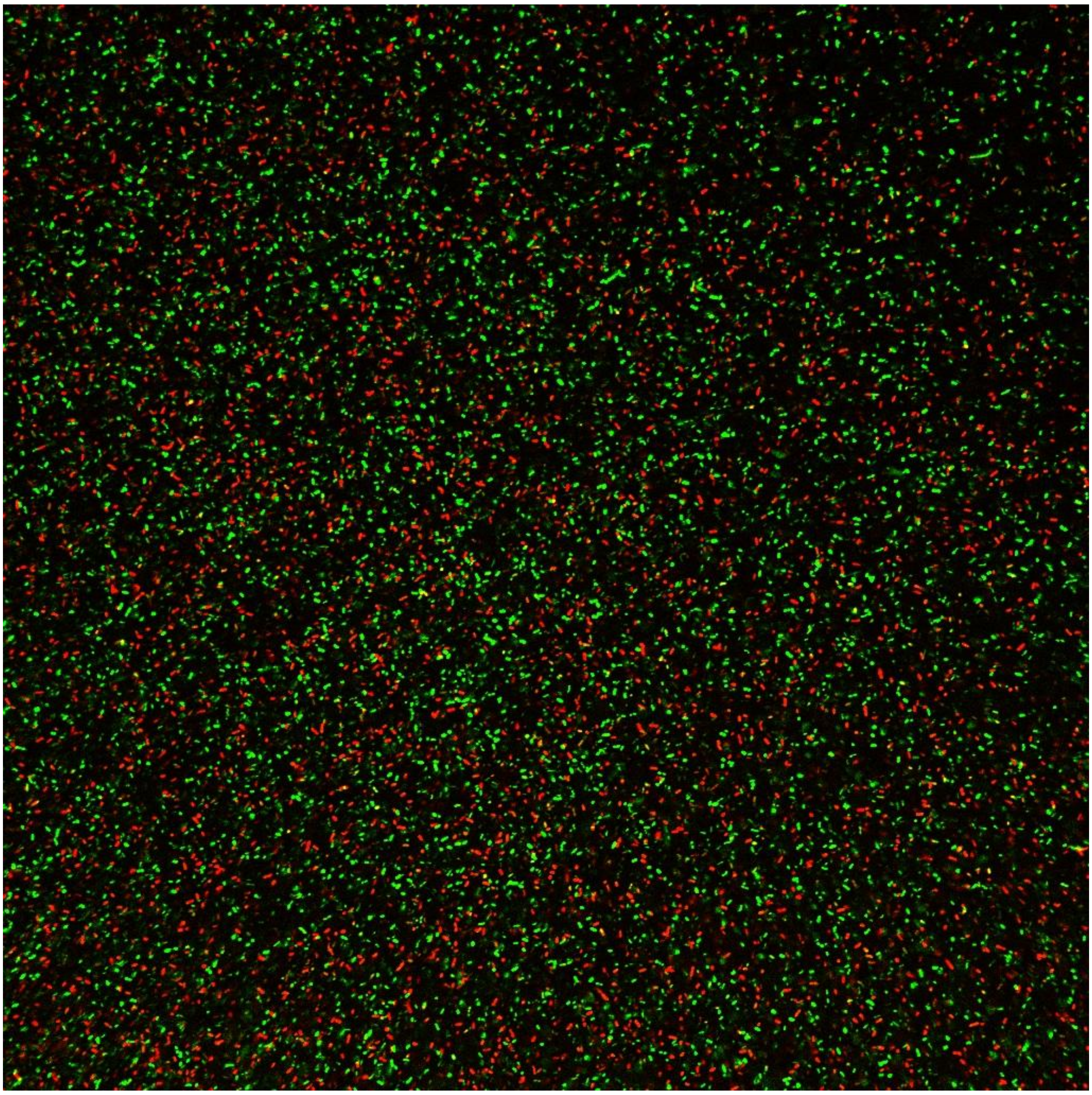

Fig. S6. Confocal laser scanning micrograph of early *P. aeruginosa* FRD1 biofilms treated with RPMI 1640 (without PMNs) for 48 hours. Biofilms were stained with SYTO 9 and propidium iodide from the LIVE/DEAD kit and observed under a Nikon A1R laser confocal microscope (Nikon, Tokyo, Japan). Cells staining red are considered dead while cells staining green are viable cells.

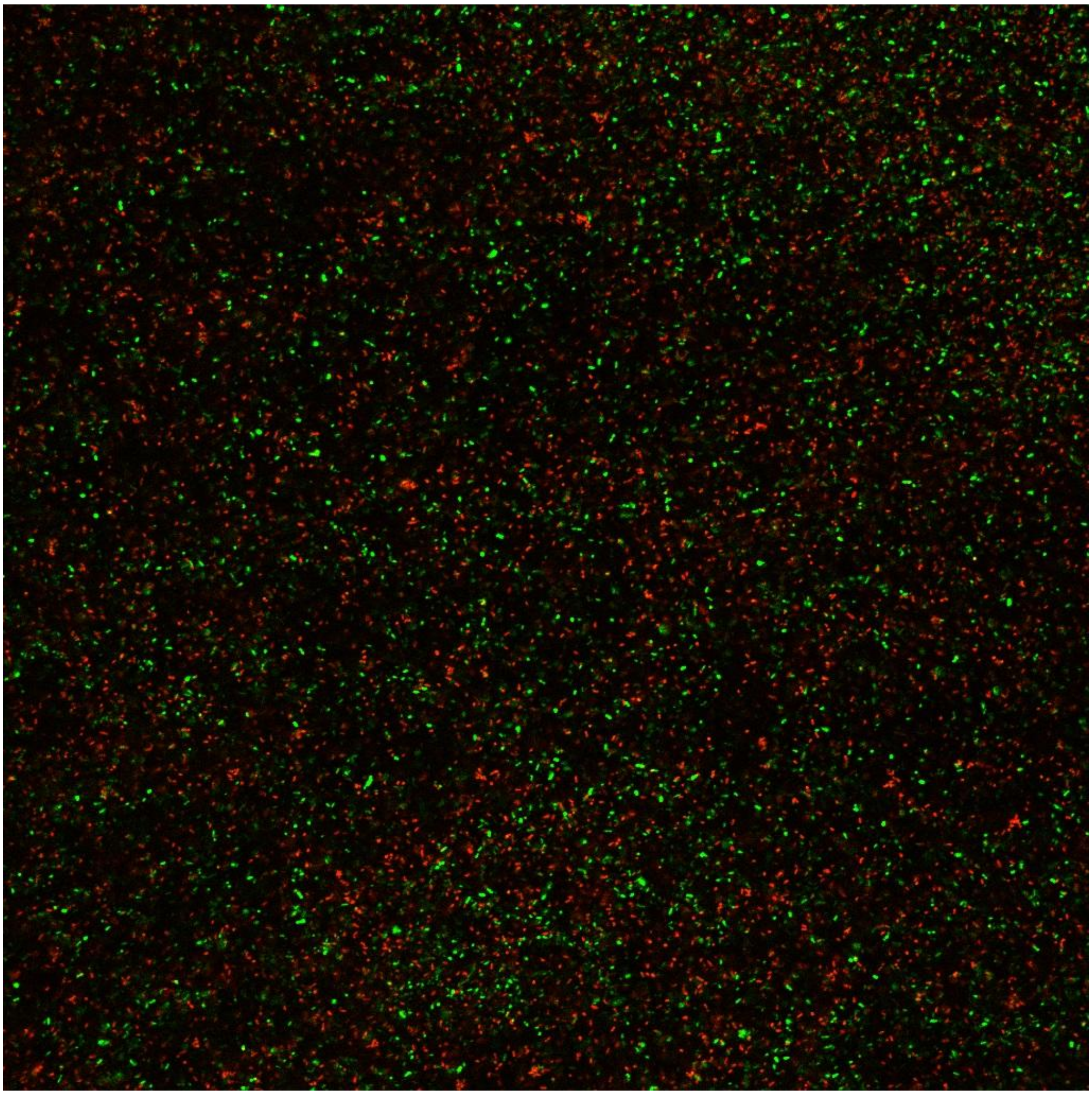

Fig. S7. Confocal laser scanning micrograph of mature *P. aeruginosa* FRD1 biofilms treated with RPMI 1640 (without PMNs) for 24 hours. Biofilms were stained with SYTO 9 and propidium iodide from the LIVE/DEAD kit and observed under a Nikon A1R laser confocal microscope (Nikon, Tokyo, Japan). Cells staining red are considered dead while cells staining green are viable cells.

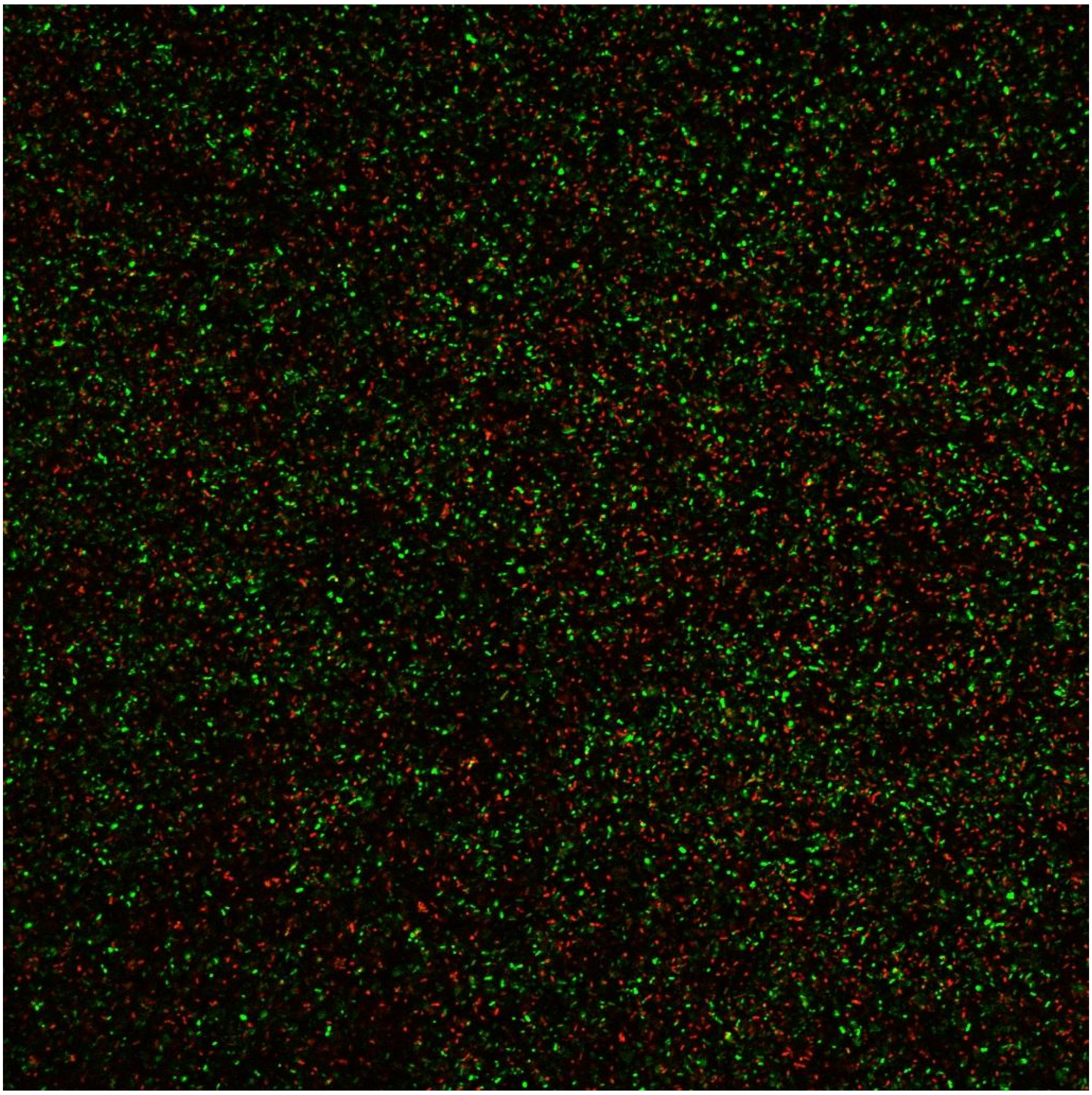

Fig. S8. Confocal laser scanning micrograph of mature *P. aeruginosa* FRD1 biofilms treated with RPMI 1640 (without PMNs) for 48 hours. Biofilms were stained with SYTO 9 and propidium iodide from the LIVE/DEAD kit and observed under a Nikon A1R laser confocal microscope (Nikon, Tokyo, Japan). Cells staining red are considered dead while cells staining green are viable cells.

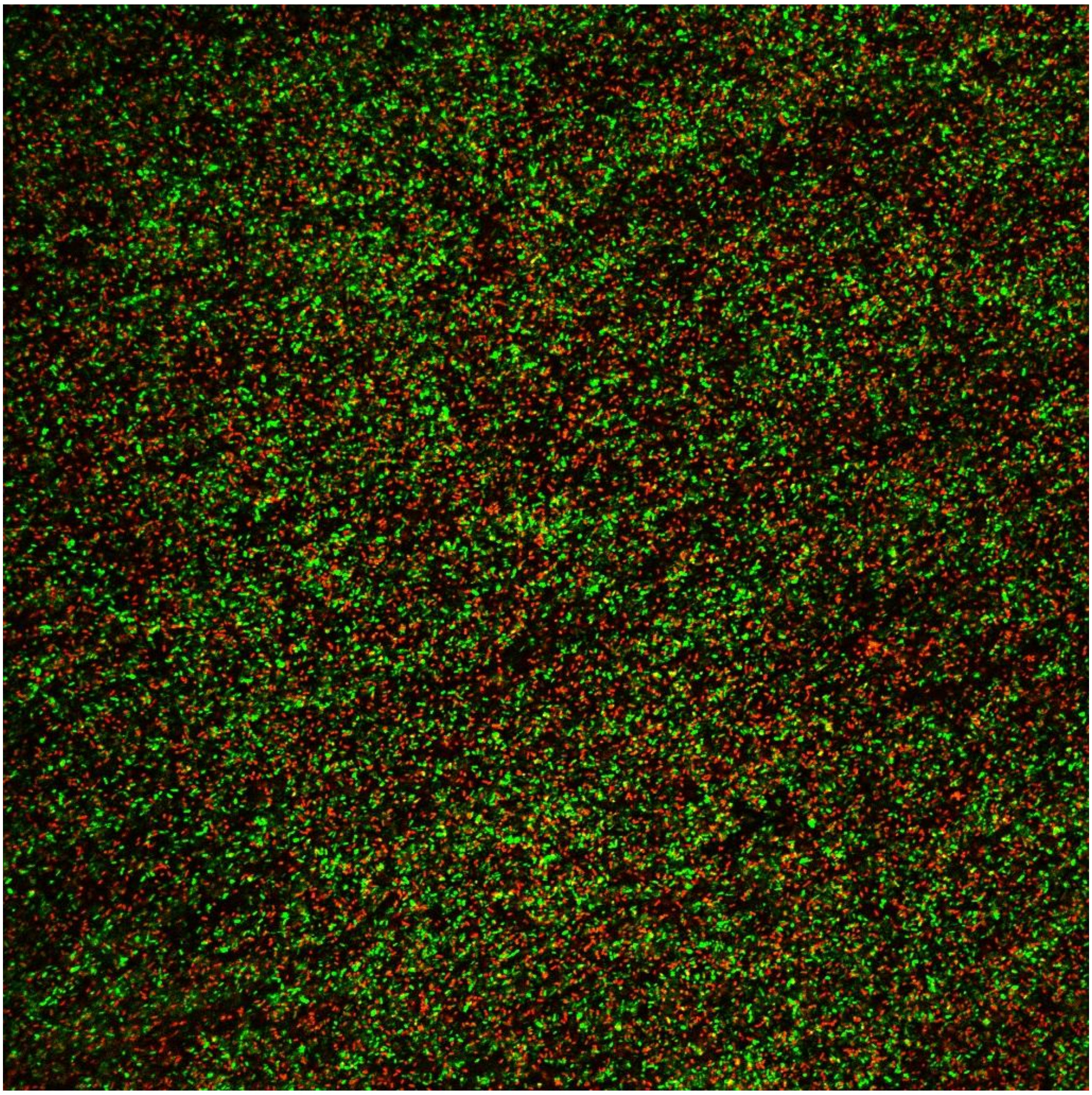

Fig. S9. Confocal laser scanning micrograph of early *P. aeruginosa* FRD1 biofilms treated with 1mM H_2O_2 for 24 hours. Biofilms were stained with SYTO 9 and propidium iodide from the LIVE/DEAD kit and observed under a Nikon A1R laser confocal microscope (Nikon, Tokyo, Japan). Cells staining red are considered dead while cells staining green are viable cells.

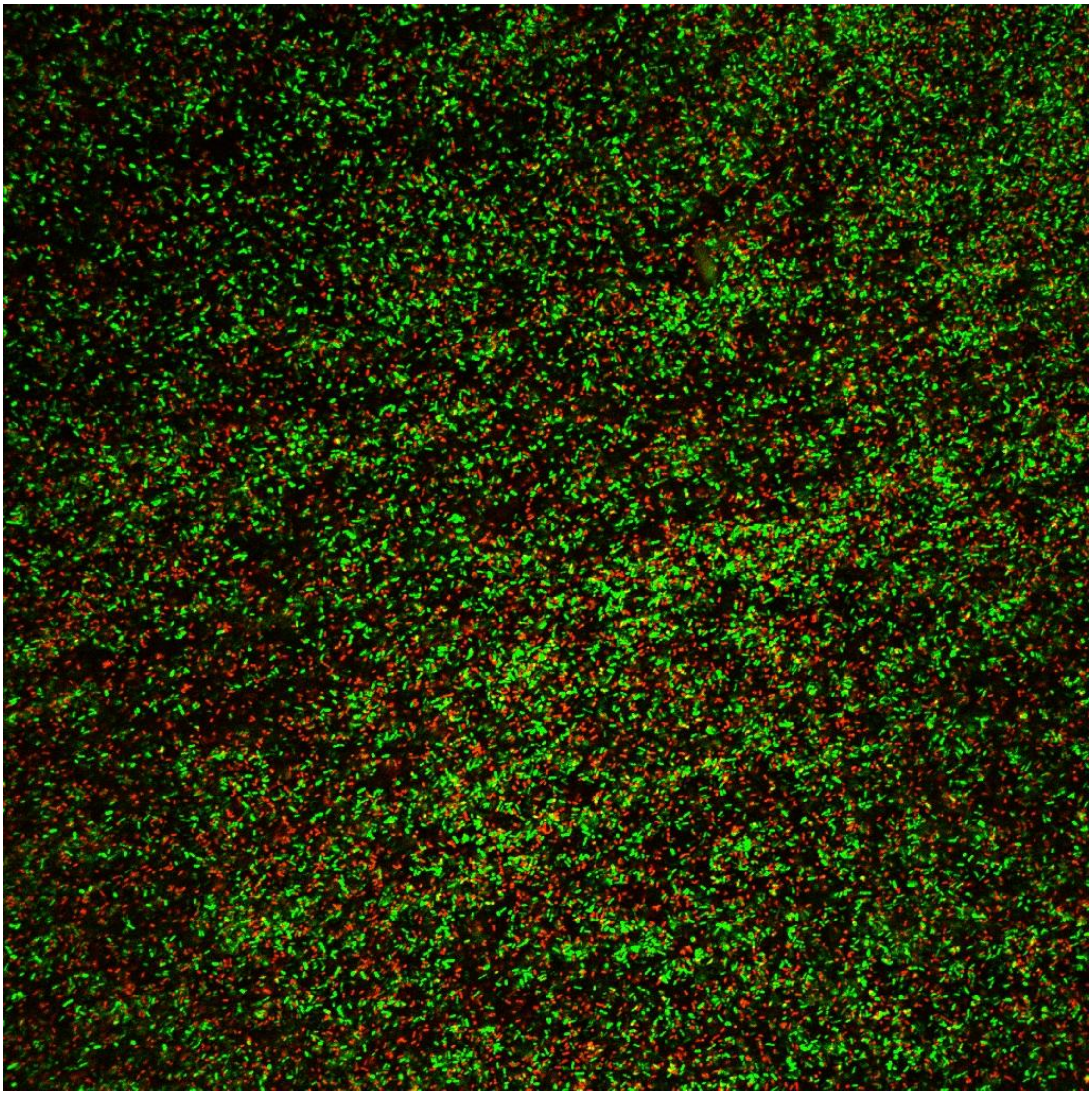

Fig. S10. Confocal laser scanning micrograph of early *P. aeruginosa* FRD1 biofilms treated with 1 mM H_2O_2 for 48 hours. Biofilms were stained with SYTO 9 and propidium iodide from the LIVE/DEAD kit and observed under a Nikon A1R laser confocal microscope (Nikon, Tokyo, Japan). Cells staining red are considered dead while cells staining green are viable cells.

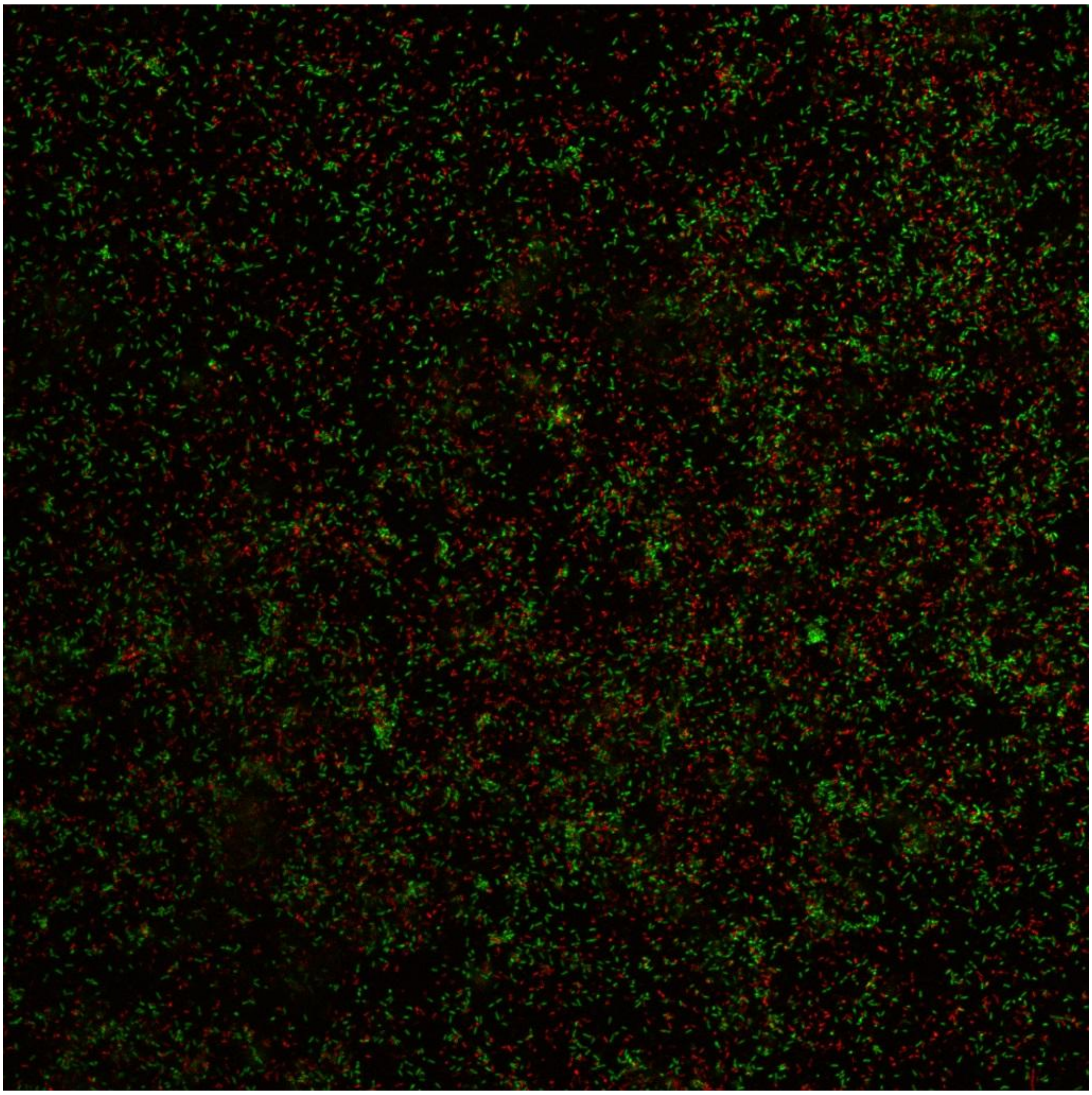

Fig. S11. Confocal laser scanning micrograph of early *P. aeruginosa* FRD1 biofilms treated with PMNs for 24 hours. Biofilms were stained with SYTO 9 and propidium iodide from the LIVE/DEAD kit and observed under a Nikon A1R laser confocal microscope (Nikon, Tokyo, Japan). Cells staining red are considered dead while cells staining green are viable cells.

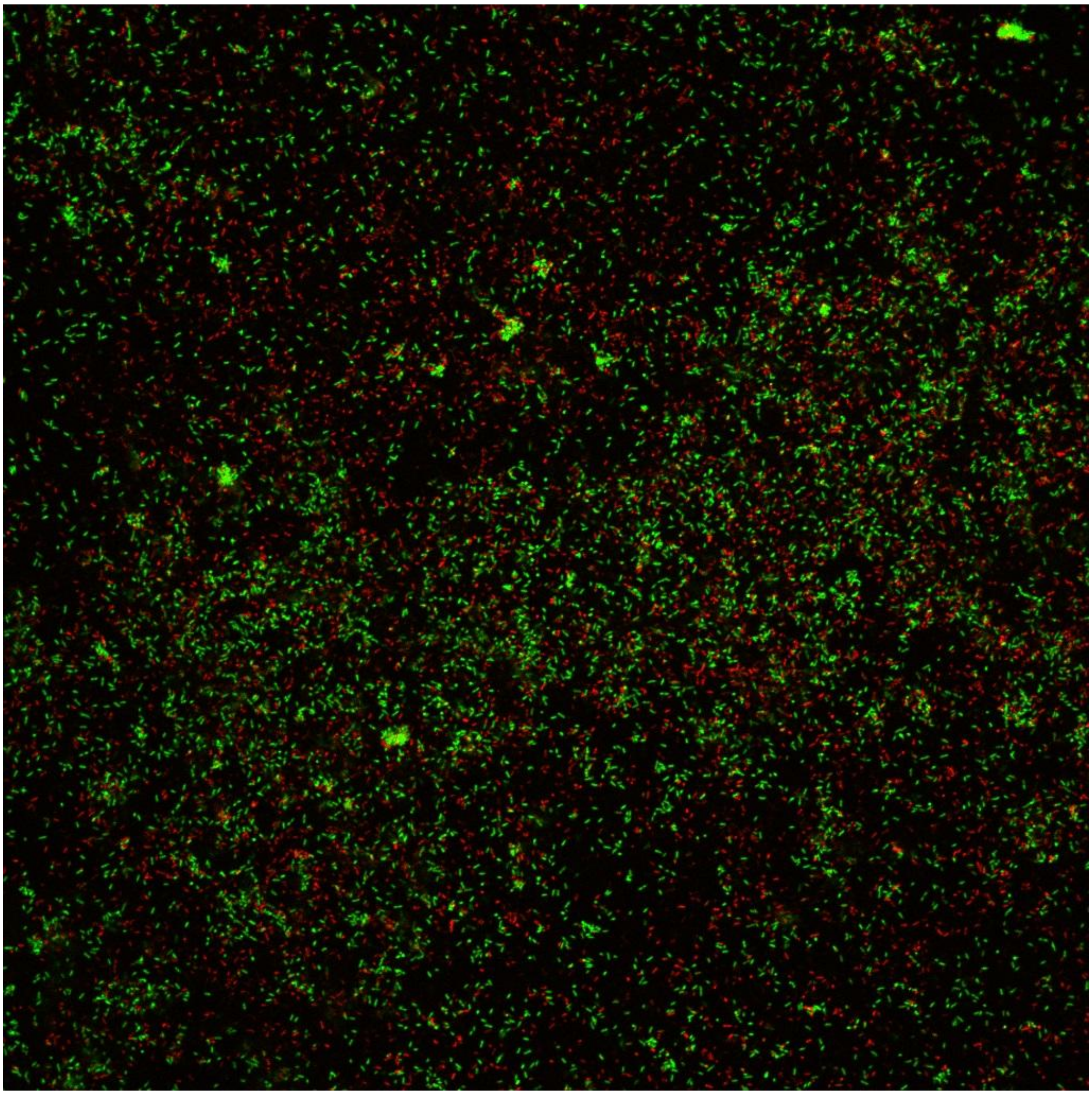

Fig. S12. Confocal laser scanning micrograph of early *P. aeruginosa* FRD1 biofilms treated with PMNs for 48 hours. Biofilms were stained with SYTO 9 and propidium iodide from the LIVE/DEAD kit and observed under a Nikon A1R laser confocal microscope (Nikon, Tokyo, Japan). Cells staining red are considered dead while cells staining green are viable cells.

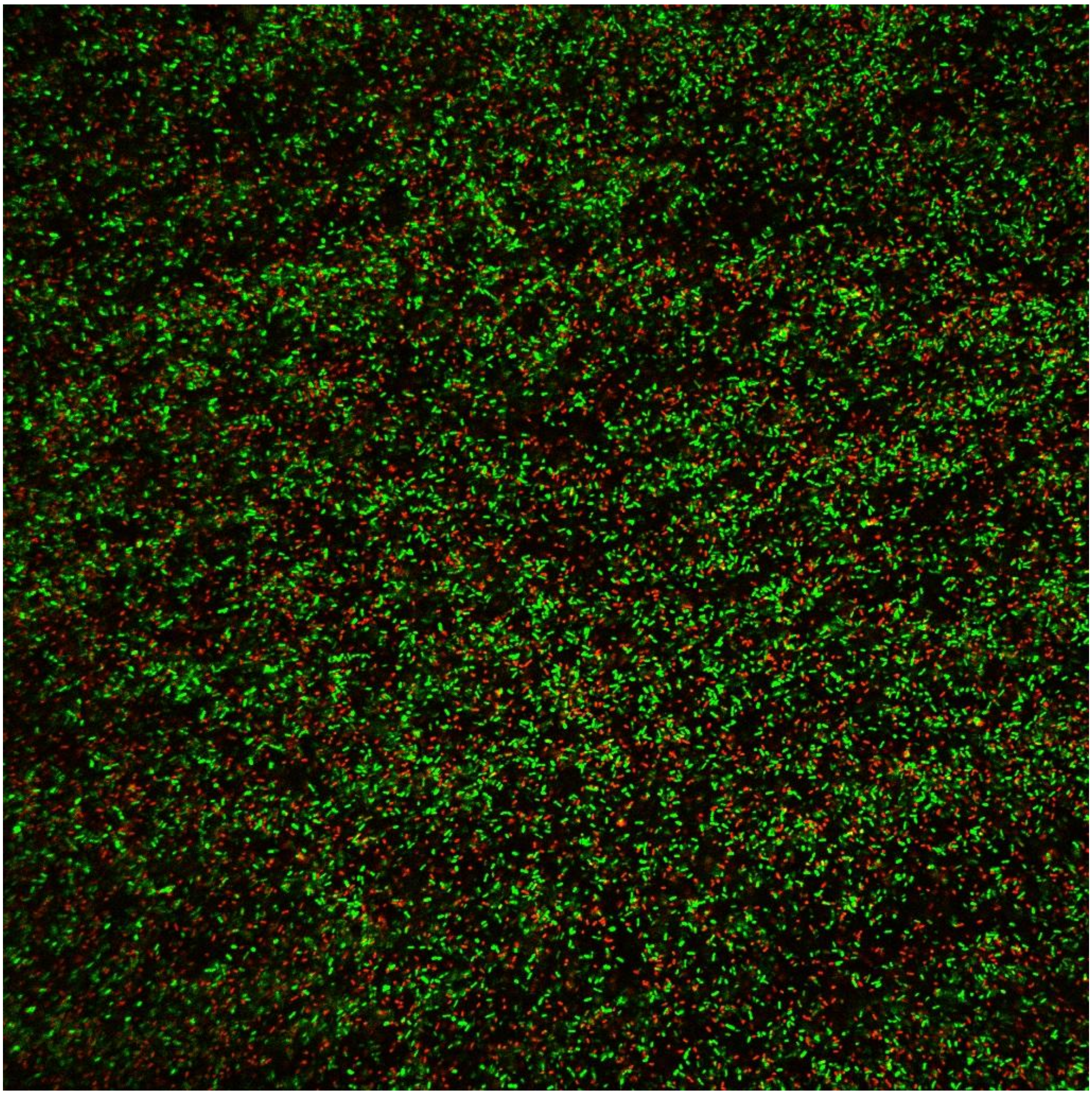

Fig. S13. Confocal laser scanning micrograph of mature *P. aeruginosa* FRD1 biofilms treated with 1 mM H_2O_2 for 24 hours. Biofilms were stained with SYTO 9 and propidium iodide from the LIVE/DEAD kit and observed under a Nikon A1R laser confocal microscope (Nikon, Tokyo, Japan). Cells staining red are considered dead while cells staining green are viable cells.

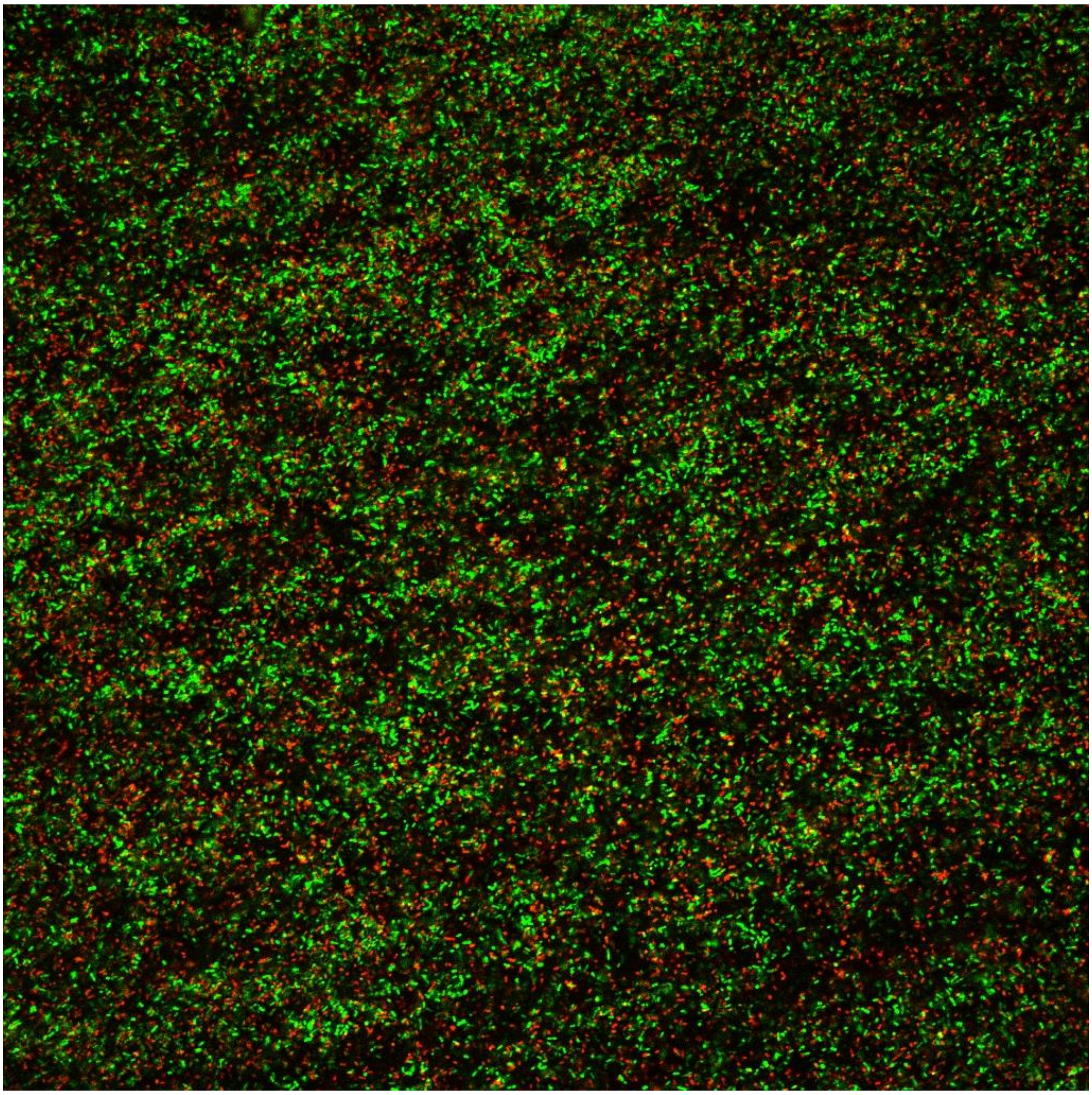

Fig. S14. Confocal laser scanning micrograph of mature *P. aeruginosa* FRD1 biofilms treated with 1 mM H_2O_2 for 48 hours. Biofilms were stained with SYTO 9 and propidium iodide from the LIVE/DEAD kit and observed under a Nikon A1R laser confocal microscope (Nikon, Tokyo, Japan). Cells staining red are considered dead while cells staining green are viable cells.

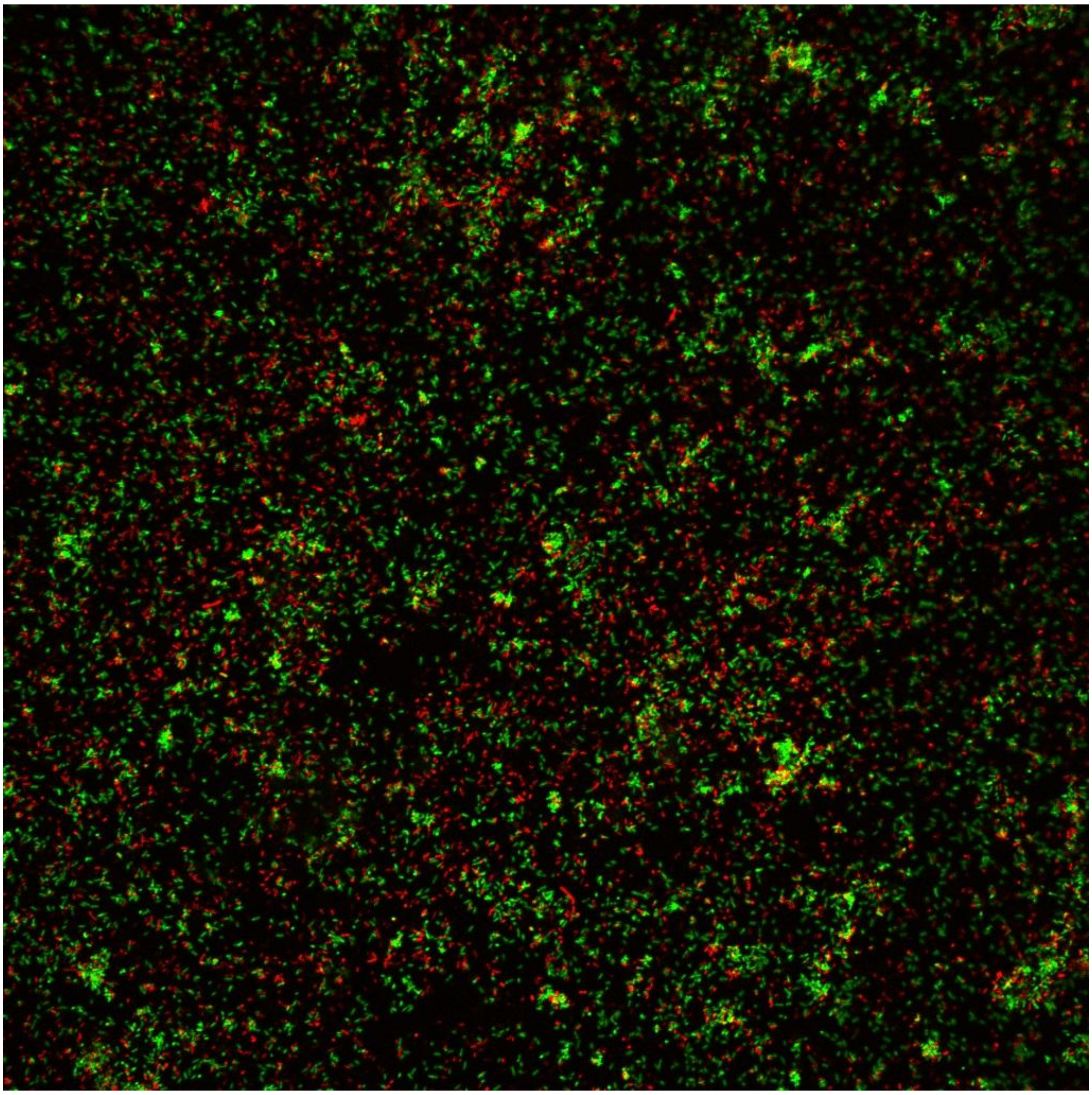

Fig. S15. Confocal laser scanning micrograph of mature *P. aeruginosa* FRD1 biofilms treated with PMNs for 24 hours. Biofilms were stained with SYTO 9 and propidium iodide from the LIVE/DEAD kit and observed under a Nikon A1R laser confocal microscope (Nikon, Tokyo, Japan). Cells staining red are considered dead while cells staining green are viable cells.
